# FGFR2 directs inhibition of WNT signaling to regulate anterior fontanelle closure during skull development

**DOI:** 10.1242/dev.204264

**Published:** 2025-01-20

**Authors:** Lauren Bobzin, Audrey Nickle, Sebastian Ko, Michaela Ince, Aaron Huang, Arshia Bhojwani, Ryan Roberts, Amy E. Merrill

**Affiliations:** Center for Craniofacial Molecular Biology, Department of Biomedical Sciences, Ostrow School of Dentistry, University of Southern California, Los Angeles, CA 90033, USA

**Keywords:** Frontal suture, FGF, Fgfr2, Scx, Anterior fontanelle, WNT, Calvaria, Lgr5, Craniofacial, Mouse

## Abstract

The calvarial bones of the infant skull are linked by transient fibrous joints known as sutures and fontanelles, which are essential for skull compression during birth and expansion during postnatal brain growth. Genetic conditions caused by pathogenic variants in *FGFR2*, such as Apert, Pfeiffer, and Crouzon syndromes, result in calvarial deformities due to premature suture fusion and a persistently open anterior fontanelle (AF). In this study, we investigated how *Fgfr2* regulates AF closure by leveraging mouse genetics and single-cell transcriptomics. We find that AF cells, marked by the tendon/ligament factor SCX, are spatially organized into ecto- and endocranial domains that selectively differentiate into ligament, bone, and cartilage to form the posterior frontal suture. We show that AF cell differentiation is non-autonomously regulated by FGFR2 signaling in osteogenic front cells of the frontal bones, which regulate WNT signaling in neighboring AF cells by expressing the secreted WNT inhibitor *Wif1*. Upon loss of *Fgfr2*, *Wif1* expression is downregulated, and AF cells fail to form the posterior frontal suture. This study identifies an FGF-WNT signaling circuit that that directs suture formation within the AF during postnatal development.

## Teaser

**Summary:** Mouse genetics and single-cell transcriptomics reveal a FGF-WNT signaling circuit that regulates closure of the anterior fontanelle, a soft spot in the infant skull.

## INTRODUCTION

The calvarial bones of the infant skull are linked by transient fibrous joints called sutures and fontanelles. Sutures, which join two adjacent calvarial bones, and fontanelles, which occupy large, unossified regions between multiple bones where sutures will eventually form, are crucial for calvarial reshaping during birth and calvarial expansion during postnatal brain growth. Numerous genetic disorders present with craniofacial deformities when sutures fuse prematurely (craniosynostosis), or fontanelles remain patent. For example, syndromes associated with genetic variants in fibroblast growth factor receptor 2 (*FGFR2*), including Apert, Pfeiffer, Crouzon, and bent bone dysplasia, often present with multi-suture craniosynostosis, as well as a persistently open anterior fontanelle (AF) (Azoury et al., 2017; Merrill et al., 2012; Reardon et al., 1994; Wilkie et al., 1995). The co-occurrence of these seemingly opposite calvarial joint phenotypes suggests important differences in the way FGF signaling regulates development of sutures versus fontanelles.

The AF, or a baby's ‘soft spot’, is a broad region of fibrous connective tissue at the apex of the developing calvaria where the paired frontal bones will ultimately meet the parietal bones. During postnatal development, the frontal bones undergo rapid apical expansion though intramembranous ossification, replacing the AF with the posterior frontal suture (PFS), which connects the frontal bones from the bregma to the jugum limitans. As the PFS forms, it differentiates into two distinct bony layers, with the endocranial layer undergoing fusion through endochondral-like ossification that requires *Sox9* and local inhibition of WNT signaling (Bradley et al., 1996; Behr et al., 2013; Manzanares et al., 1988; Sahar et al., 2005). The fate of the AF connective tissue and its role in frontal suture development, as well as its potential contribution to the etiology of calvarial defects, has remained unclear.

While calvarial phenotypes in *FGFR2*-related syndromes indicate a key role for FGF signaling in the developing AF, studies to date have largely focused on the role of the receptor in bone formation during embryogenesis when it is expressed within the mid-suture and advancing osteogenic bone fronts. Genetic studies in mice have shown that FGFR2, likely activated by FGF18, is crucial for maintaining the balance of proliferation and osteoblast differentiation within sutures (Ohbayashi et al., 2002). In mouse models that harbor gain-of-function *Fgfr2* variants associated with human craniosynostosis syndromes, mechanisms of premature fusion of the coronal suture include altered proliferation and induction of ectopic osteoblast differentiation (Holmes et al., 2009; Wang et al., 2005; Yin et al., 2008; Pfaff et al., 2016; Hajihosseini et al., 2001). These mouse models also exhibit a midline gap between the frontal bones in a region anatomically homologous to the human AF. Studies using the Apert syndrome mouse model have shown that this midline gap is linked to decreased osteoblast differentiation, as well as transcriptomic changes in the cells within the osteogenic fronts of the frontal bones (Wang et al., 2005; Holmes et al., 2020). A midline ossification defect was also reported in mice with conditional loss of *Fgfr2* in skeletogenic mesenchyme, but the mechanism underlying the phenotype has yet to be investigated (Yu et al., 2003).

Here, we show that *Fgfr2* is necessary for AF closure and, subsequently, frontal suture joint formation. We find that cells of the AF express the tendon/ligament marker *Scx* and represent at least two transcriptionally distinct populations spatially restricted to either the ecto- or endocranial domains, which give rise to bone and cartilage, respectively, in an FGF-dependent manner. We provide evidence that FGF signaling in the osteogenic fronts of the frontal bone promotes expression of the secreted WNT inhibitor *Wif1*, which non-autonomously regulates WNT signaling in AF cells. In the absence of FGF signaling, *Wif1* expression is lost, and cells of the AF fail to differentiate into bone and cartilage, instead maintaining a connective tissue-like identity. Correspondingly, elevation of WNT/β-catenin signaling induces a persistent AF phenotype, and downregulation of WNT/β-catenin rescues AF closure in *Fgfr2* mutant mice. Together, these results suggest that FGF-mediated regulation of WNT signaling regionally influences cell fate and suture formation within the AF.

## RESULTS

### *Fgfr2* is necessary for closure of the AF

**Fig. 1.**
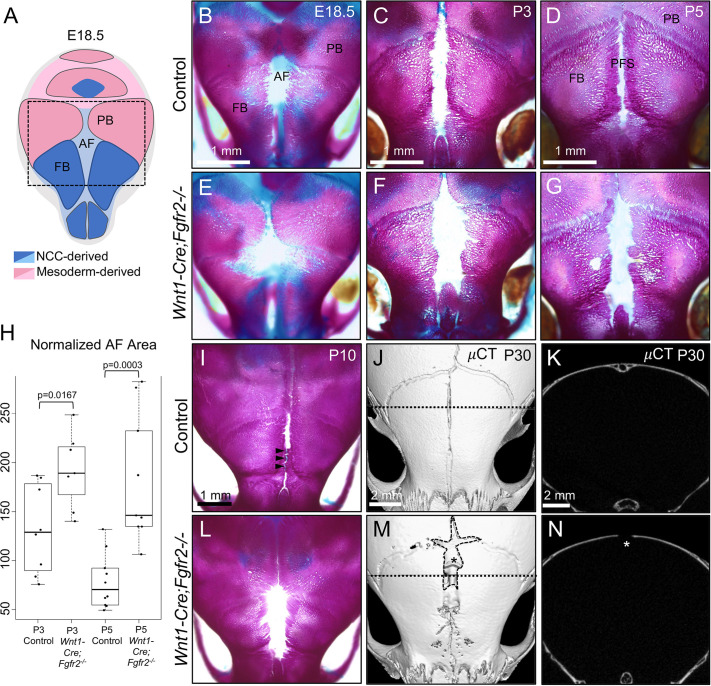
***Fgfr2* is required in NCC-derived mesenchyme for closure of the AF and formation of PFS.** (A) Diagram representing the tissue origins of the calvarial vault. NCC-derived regions are depicted in blue and paraxial mesoderm-derived regions are depicted in pink. Darker shades of either color represent bone while lighter shades represent fibrous connective tissue, including the fontanelle. (B-G) Wholemount skeletal preparation staining using Alizarin Red (bone) and Alcian Blue (cartilage) showing morphological differences between the AF of controls and *Wnt1-Cre;Fgfr2^−/−^* littermates throughout development. (B,E) At E18.5, *Wnt1-Cre;Fgfr2^−/−^* mice showed a slightly wider anterior fontanelle compared to control. (C,D,F,G) By P3 and P5, the difference became more pronounced as the frontal bones of *Wnt1-Cre;Fgfr2^−/−^* mutants failed to approximate. (H) Quantification of the normalized AF area at P3 and P5 showed statistically significant differences between control and *Wnt1-Cre;Fgfr2^−/−^* mice. The box represents the upper and lower quartiles, with the median indicated by a horizontal line. Each dot represents an individual sample and the whiskers represent the range (highest and lowest) measurements in the group. (I,L) At P10, the control PFS began to fuse anteriorly, forming cartilage adjacent to the jugum limitans, while *Wnt1-Cre;Fgfr2^−/−^* mutants completely lacked this cartilage as frontal bones still failed to advance. (J,K,M,N) μCT scans of *Wnt1-Cre;Fgfr2^−/−^* mutant and littermate control at P30 showed a persistent AF (marked with asterisk) and dysmorphia of the anterior PFS. Dotted lines in J and M correspond to orthoslices that showed lack of frontal suture formation (asterisk; K,N). AF, anterior fontanelle; FB, frontal bone; PFS, posterior frontal suture; PB, parietal bone. At least seven littermate pairs were analyzed for each stage.

Genetic lineage analysis in mice using *Wnt1-Cre* previously showed that neural crest-derived mesenchyme gives rise to the frontal bones, as well as the tissue that occupies the AF (Fig. 1A) (Jiang et al., 2002; Yoshida et al., 2008). Therefore, to investigate a role of FGFR2 within the AF development, we performed *Wnt1-Cre*-mediated deletion of *Fgfr2* using a floxed allele that produces a functional null copy of the receptor (Yu et al., 2003). We examined calvarial bone and cartilage development in *Wnt1-Cre;Fgfr2^−/−^* and littermate controls using wholemount Alizarin Red and Alcian Blue staining. The AF of *Wnt1-Cre;Fgfr2^−/−^* mice was wide and patent beginning at embryonic day (E) 18.5 compared to control (Fig. 1B,E). In control mice at postnatal day (P) 3, the AF began to progressively close, whereas that of *Wnt1-Cre;Fgfr2^−/−^* mice remained wide open (Fig. 1C,F). By P5, the frontal bones of control mice had closely approximated to form the PFS, whereas the AF remained persistent in the mutant (Fig. 1D,G). Quantitative measurement showed that the normalized average area between the frontal bones (total area of AF/length of PFS from jugum limitans to bregma) was significantly larger in *Wnt1-Cre;Fgfr2^−/−^* at P3 (*P*=0.0167, *n*=7) and P5 (*P*=0.0003, *n*=9) compared to littermate controls (Fig. 1H). By P10, the PFS of control mice began to form cartilaginous fusions that follow a joint-gap pattern (Fig. 1I, arrowheads), whereas the AF failed to resolve into the PFS in *Wnt1-Cre;Fgfr2^−/−^* mice (Fig. 1L). Micro-computed tomography (μCT) analysis showed that the PFS of control mice fused by P30, with distinct endocranial and ectocranial layers visible in orthoslice views (Fig. 1J,K). In *Wnt1-Cre;Fgfr2^−/−^* mice, the AF persisted, and regions of the frontal and sagittal sutures that it prefigures failed to form (Fig. 1M,N). Together, these results show that *Fgfr2* is necessary for AF closure.

### Loss of *Fgfr2* blocks posterior frontal suture formation within the AF

**Fig. 2.**
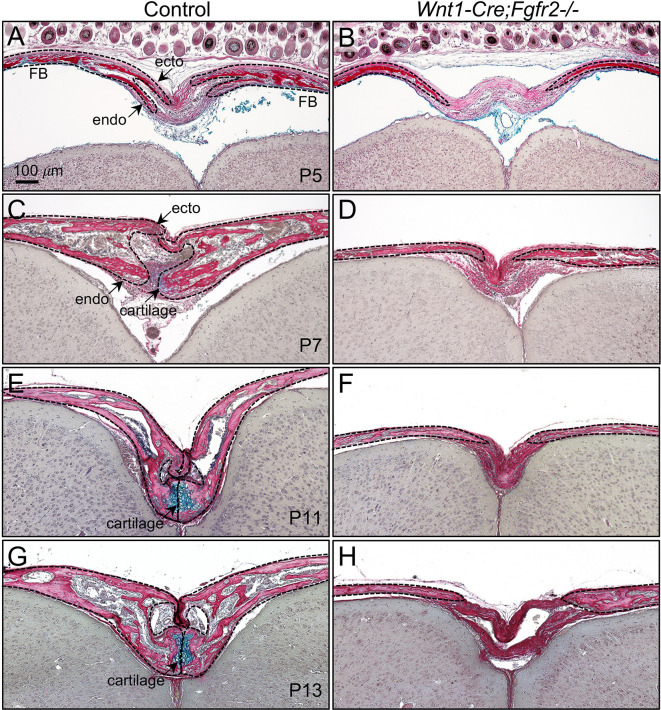
***Fgfr2* is required in NCC-derived mesenchyme for closure of the AF.** (A-H) HBQ-stained coronal sections at crucial stages of PFS development. (A,B) The ectocranial (ecto) and endocranial (endo) layers of the PFS formed in controls at P5, but only the frontal bone fronts were identified in *Wnt1-Cre;Fgfr2^−/−^* mutants. (C,D) At P7, when cartilage (blue) begins to condense in the endocranial layer of the PFS in control, there was no evidence of cartilage in *Wnt1-Cre;Fgfr2^−/−^* mice. (E-H) At P11 and P13, when the endocranial cartilage matures and begins to ossify in control, there remained no cartilage in *Wnt1-Cre;Fgfr2^−/−^* mutants and the frontal bones were separated by connective tissue. At least four littermate pairs were analyzed per stage. Dashed lines outline frontal bone and osteogenic fronts.

The posterior frontal suture (PFS), which forms within the AF, undergoes normal fusion in the endocranial domain through a cartilage intermediate. To identify histogenic changes in PFS development, Hall-Brunt Quadruple stain (HBQ) was used to distinguish bone and cartilage (Hall, 1986). In control mice at P5, the paired frontal bones approximated in the ectocranial domain and secondary osteogenic fronts formed within the endocranial domain (Fig. 2A). The frontal bones of *Wnt1-Cre;Fgfr2^−/−^* mice, however, remained far apart and the secondary osteogenic fronts within the endocranial domain were missing (Fig. 2B). In control mice at P7, the cartilage template that prefigures the fused endocranial layer of the PFS began to condense, becoming more differentiated at P11 and P13 (Fig. 2C,E,G). Conversely, the frontal bones of *Wnt1-Cre;Fgfr2^−/−^* mice remained separated and showed no evidence of cartilage formation in the endocranial domain (Fig. 2D,F,H). Together, these results show that *Fgfr2* is necessary for normal formation and fusion of the PFS within the AF.

### *Fgfr2* regulates cell identity in the AF

**Fig. 3.**
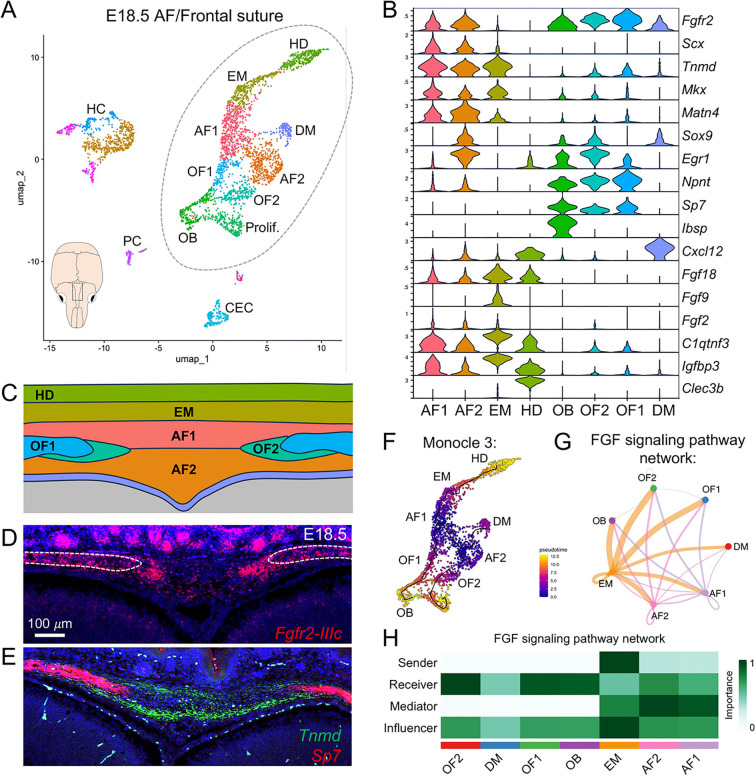
**scRNA-seq identifies heterogeneous cell populations within the developing AF.** (A) Uniform manifold approximation and projection (UMAP) visualization for scRNA-seq of the E18.5 frontal suture. Mouse skull diagram with boxed region highlights the tissue collected for analysis. Dashed circle outlines the mesenchymal cell types that were more closely delineated in our analysis. (B) Violin plots showing mesenchymal clusters enriched for *Fgfr2* expression along with cluster-specific gene expression. (C) Diagram of the developing AF showing the precise locations of each mesenchymal cell cluster based on our analysis and a previous study that described this dataset (Holmes et al., 2020). Colors coordinate with cluster identities shown in A and B. (D,E) *In situ* hybridization validated expression of *Fgfr2-IIIc* (red) in OF1 and OF2, marked by *Sp7* (red), and AF1 and AF2, marked by *Tnmd* (green), in the AF at E18.5. Dashed lines in D outline osteogenic fronts. (F) Pseudotime analysis performed with Monocle 3 predicted that cells in AF1 and AF2 clusters differentiate into OF1 and OF2, respectively. (G,H) CellChat analysis predicted that the ectocranial mesenchyme (EM) is a source of FGFs that signal to FGFR-expressing cells in the anterior fontanelle (AF1 and AF2 clusters) and the osteogenic front (OF1 and OF2 clusters), with the osteogenic cells (OF1, OF2, and OB) being the primary receivers. AF, anterior fontanelle; CEC, capillary endothelial cells; DM, dura mater; EM, ectocranial mesenchyme; HC, hematopoietic cells; HD, hypodermis; OB, osteoblasts; OF, osteogenic front; PC, pericytes; Prolif., proliferating osteoblasts.

To better understand *Fgfr2*^+^ cell populations in the developing AF, we analyzed a previously published single-cell RNA sequencing (scRNA-seq) dataset of the developing frontal suture at E18.5, which included the paired osteogenic fronts of the frontal bones and intervening AF connective tissue (FaceBase dataset 1-4TT6; Holmes et al., 2020). Re-analysis of this dataset using Seurat identified nine mesenchymal clusters with similar gene enrichment to those previously reported (Fig. 3A,C) (Holmes et al., 2020). Violin plots showed enriched *Fgfr2* expression in cell clusters we designated AF1 and AF2 based on their expression of genes associated with ligament-like connective tissue, including *Scx*, *Tnmd*, *Mkx*, and *Matn4* (Fig. 3B). Interestingly, AF2 was distinct from AF1 based on its enriched expression of the chondrogenic gene *Sox9* and tenogenic gene *Egr1* (Fig. 3B). This is consistent with a previous study that showed *Sox9* to be indispensable for closure of the PFS through endochondral-like ossification (Sahar et al., 2005). *Fgfr2* expression was most enriched in the osteogenic populations, including the osteoblasts (OB) cluster, marked by *Ibsp* expression, and the osteogenic front (OF) 1 and 2 clusters, which expressed pro-osteogenic genes *Npnt* and *Sp7* (Fig. 3B). Interestingly, *Sp7* has been implicated in regulating cellular recruitment at advancing bone fronts (Kague et al., 2016; Wang et al., 2022). Compared to OF1, the OF2 cluster was enriched for AF2-related genes including *Sox9* and *Egr1*. Relatively lower levels of *Fgfr2* were expressed in the dura mater (DM) cluster, which was marked by expression of *Cxcl12* (Fig. 3B) (Holmes et al., 2020). While *Fgfr2* expression was not enriched in the ectocranial mesenchyme (EM) cluster, marked by *C1qtnf3* and *Igfbp3*, or the hypodermis (HD) cluster, marked by *Clec3b*, these clusters expressed the FGFR2 ligand *Fgf18* (Fig. 3B) (Farmer et al., 2021). *Fgf9* and *Fgf2*, the only other FGF ligands detected in the dataset, were also enriched in the EM cluster. RNAScope fluorescent *in situ* hybridization at E18.5 validated that the spatial distribution of *Fgfr2-IIIc* (the mesenchymal-specific variant of *Fgfr2*) expression overlapped with expression domains for the AF1 and AF2 marker *Tnmd* and the OF1 and OF2 marker *Sp7* (Fig. 3D,E). Pseudotime analysis performed with Monocle 3 predicted that the AF1 and AF2 clusters follow distinct developmental trajectories to give rise to OF1 and OF2, respectively, and converge on an osteogenic identity (Fig. 3F) (Cao et al., 2019). This likely represents the distinct contribution of *Sox9*^+^ AF2 cells to endochondral-like bone that forms within the endocranial domain of the PFS. Cellular communication modeling using CellChat indicated that FGFs expressed in the EM signal to FGFR-expressing cells in the AF and OF populations (Fig. 3G,H) (Jin et al., 2021).

**Fig. 4.**
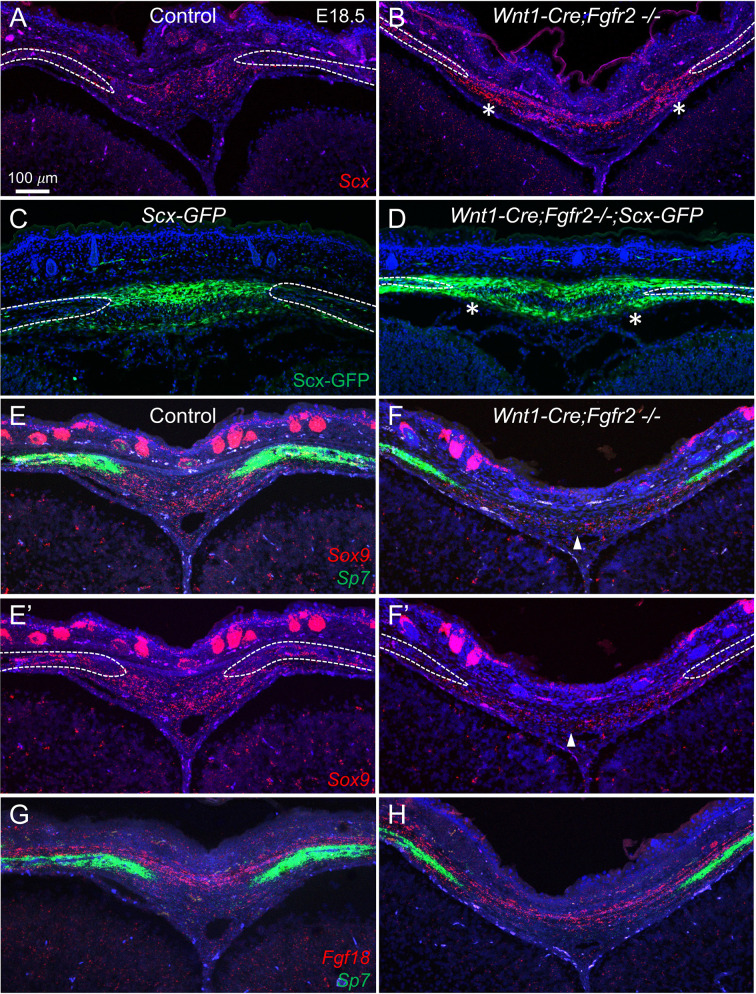
**Expression of cell type-specific genes are altered in AF of *Wnt1-Cre;Fgfr2^−/−^* embryos.** (A-H) *In situ* hybridization with RNAScope was used to identify changes in cluster-specific gene expression in the AF at E18.5. (A,B) *Scx*, a marker for AF1 and AF2, was expressed within the AF in control. This expression domain was expanded towards the osteogenic fronts in the *Wnt1-Cre;Fgfr2^−/−^* mutant AF (asterisks). (C,D) A similar expansion of Scx-GFP was seen in the *Wnt1-Cre;Fgfr2^−/−^* mutant AF compared to control (asterisks). (E,E′) In control, *Sox9*, a marker for AF2 and to a lesser extent OF2, was localized to the endocranial domain of the AF and within the bone. *Sp7*, a marker for OF1 and OF2, was expressed throughout the bone. (F,F′) In the *Wnt1-Cre;Fgfr2^−/−^* AF, *Sox9* expression was reduced within the AF (arrowhead) and largely missing from the bone labeled with *Sp7.* (G,H) The domain of *Fgf18* expression in the EM showed little to no difference between control and *Wnt1-Cre;Fgfr2^−/−^* mutant AF relative to the osteogenic fronts marked by *Sp7* expression. Lines in E′ and F′ indicate the domain of *Sp7* expression in E and F. Dashed lines in other panels delineate the frontal bones. *n*=3 littermate pairs per stage.

To determine how loss of *Fgfr2* impacts the cell populations identified by scRNA-seq, we examined changes in cluster-enriched gene expression in the developing AF at E18.5 using RNAScope *in situ* hybridization. In the control, *Scx*, which marks the AF1 and AF2 clusters, was expressed in the AF connective tissue between the bone fronts with highest expression in the ectocranial region (Fig. 4A). The expression domain of *Scx* expression was expanded in *Wnt1-Cre;Fgfr2^−/−^* littermates, likely indicative of the increased size of the AF in mutants (Fig. 4B, asterisks). An expanded domain of Scx-GFP expression was also seen at this stage in *Wnt1-Cre;Fgfr2^−/−^* compared to controls (Fig. 4C,D, asterisks). *Sp7*, a marker for the OF1 and OF2 clusters, was expressed in osteogenic fronts of controls and reduced in *Wnt1-Cre;Fgfr2^−/−^* littermates (Fig. 4E,F,G,H). In control, *Sox9*, a marker for the AF2 and OF2 clusters, was expressed in the endocranial region of the AF and within the osteogenic fronts (Fig. 4E,E′). Expression of *Sox9* was reduced in the AF and osteogenic fronts of *Wnt1-Cre;Fgfr2^−/−^* littermates (Fig. 4F,F′, arrowhead). By contrast, no difference in the expression of *Fgf18*, a marker for the EM cluster, was detected (Fig. 4G,H). Together, these results provide evidence that transcriptionally distinct populations of AF cells occupy spatially separate domains, and their markers are differentially impacted by loss of *Fgfr2*.

### *Fgfr2* is required for osteogenic differentiation of AF cells

Very little is known about the AF connective tissue cells and their role in frontal suture formation. To observe AF connective tissue cells during calvarial development, we employed a Scx-GFP transgenic reporter mouse that has been previously used to study tendon and ligament development (Pryce et al., 2007). At E18.5, Scx-GFP was expressed in all calvarial sutures and fontanelles, as well as in the immediately adjacent bone (Fig. S1A,C). At P3, when the frontal suture had largely replaced the AF, Scx-GFP expression was confined to the suture connective tissue (Fig. S1B,D). Sections through the AF at these stages revealed a bi-layered expressional pattern of Scx-GFP representing the ecto- and endocranial layers of the AF at E18.5 and P3, as well as some GFP^+^ cells observed within the frontal bones at P3 (Fig. S1E,F). At P3, Scx-GFP expression in the ectocranial layer likely marked the developing suprasutural ligament.

**Fig. 5.**
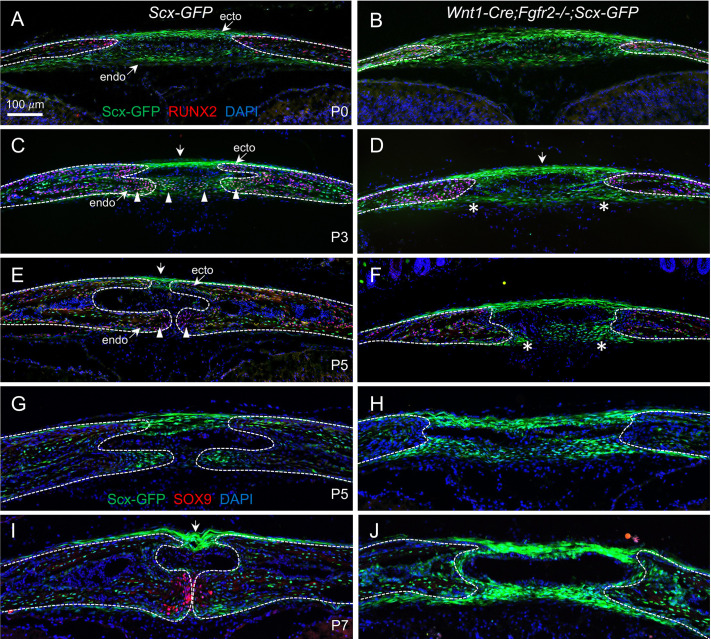
***Fgfr2* is necessary for differentiation of SCX^+^ cells of the AF into skeletogenic cells expressing RUNX2 and SOX9 during PFS formation.** (A,B) Scx-GFP (green) expression along with immunofluorescence labeling of the preosteoblast marker RUNX2 (red) showed that SCX^+^ cells within the AF are non-osteogenic in control and *Wnt1-Cre;Fgfr2^−/−^* mice at P0. (C,D) In control at P3, during PFS formation, SCX^+^ cells within the AF began to express RUNX2 (SCX^+^/RUNX2^+^) (arrowheads). A subset of SCX^+^ cells in the ectocranial domain remained RUNX2 negative (arrow). RUNX2 remained undetectable in SCX^+^ cells within the *Wnt1-Cre;Fgfr2^−/−^* AF (asterisks). (E,F) In control at P5, after the PFS is established, most SCX^+^ cells expressed RUNX2 (arrowheads) with the exception of SCX^+^ cells just above the suture that will form the suprasutural ligament (arrow). In *Wnt1-Cre;Fgfr2*^−/−^ mice, most SCX^+^ cells in the endocranial domain remain RUNX2 negative (asterisks). (G,H) Scx-GFP (green) expression along with immunofluorescence labeling of the chondrocyte marker SOX9 (red) identified no SOX9^+^ cells in the PFS of control or *Wnt1-Cre;Fgfr2^−/−^* mice at P5. (I,J) At P7, SCX^+^ cells began expressing SOX9 (SCX^+^/SOX^+^) within the endocranial domain of the control PFS, whereas SOX9^+^ cells failed to form in the *Wnt1-Cre;Fgfr2^−/−^* PFS. Arrow indicates a suprasutural ligament. Dashed lines in each panel outline osteogenic fronts. *n*=3 littermate pairs per stage.

We next examined osteogenic differentiation within the AF of *Wnt1-Cre;Fgfr2^−/−^* mice using immunofluorescent detection of RUNX2, an early marker of osteoblasts, along with Scx-GFP to discriminate the AF connective tissue. At P0, RUNX2^+^ cells were enriched within the osteogenic fronts of the paired frontal bones, and Scx-GFP expression was localized to cells in the ecto- and endocranial layers of the AF in both control and *Wnt1-Cre;Fgfr2^−/−^* mice (Fig. 5A,B). In control mice at P3, RUNX2 expression was activated in SCX^+^ cells located in the endocranial layer of the AF connective tissue, and SCX^+^/RUNX2^+^ double-positive cells were found in the advancing frontal bones (Fig. 5C, arrowheads). SCX^+^/RUNX2^+^ cells failed to form in the endocranial layer of *Wnt1-Cre;Fgfr2^−/−^* mice, remaining exclusively SCX^+^ within the AF (Fig. 5D, asterisks). The ectocranial layer of SCX^+^ cells spanning and overlaying the frontal bones at this stage remained similar between control and mutant mice (Fig. 5C,D, arrows). In controls at P5, RUNX2^+^ cells coalesced at the approximating endocranial osteogenic fronts to form the PFS, and SCX^+^/RUNX2^+^ cells showed contribution to the approximating bone fronts (Fig. 5E, arrowheads). Meanwhile, a portion of the ectocranial layer in control mice remained occupied by SCX^+^ connective tissue that will become the suprasutural ligament **(**Fig. 5E, arrow**)**. Conversely, in *Wnt1-Cre;Fgfr2^−/−^* mice the AF remained patent and very few SCX^+^/RUNX2^+^ were detected in the osteogenic fronts or bones (Fig. 5F, asterisks).

To detect chondrogenic differentiation, we performed immunofluorescent detection of SOX9, an early marker for chondrocytes. SOX9 was not detected in the PFS at P5 in control or *Wnt1-Cre;Fgfr2^−/−^* mice (Fig. 5G,H), but a population of SOX9^+^ cells appeared in the endocranial region of the suture midline in controls by P7 (Fig. 5I). At this stage, the SCX^+^ suprasutural ligament was clearly distinguishable (Fig. 5I, arrow). In *Wnt1-Cre;Fgfr2^−/−^* mice, however, SOX9 expression remained undetectable, and the persistent AF was occupied by SCX^+^ cells that structurally resembled suprasutural ligament in both the ecto- and endocranial layers (Fig. 5J). We then investigated whether the loss of SOX9^+^ cells could be caused by cell death in the *Wnt1-Cre;Fgfr2^−/−^* mice. Immunofluorescence for cleaved-caspase showed no cell death in controls or *Wnt1-Cre;Fgfr2^−/−^* mutants at P0, P3, or P5 (Fig. S2). Together, these results strongly indicate that the SCX^+^ cells of the AF connective tissue normally undergo regionally selective differentiation to contribute to intramembranous bone, endochondral bone, and ligament in the PFS, and that the persistent AF in *Wnt1-Cre;Fgfr2^−/−^* mice is largely caused by failed differentiation of SCX^+^ cells into RUNX2^+^ and SOX9^+^ skeletogenic cells.

*Fgfr2* has a well-established role in regulating proliferation and differentiation in the developing sutures. We next investigated how loss of *Fgfr2* impacts proliferation during AF closure using 5-ethynyl-2-deoxyuridine (EdU) pulse-chase experiments, since persistence of the AF in *Wnt1-Cre;Fgfr2^−/−^* mice could be due to increased proliferation in the AF and/or decreased proliferation in the osteogenic fronts of the frontal bone. At E18.5, no statistically significant differences were identified in the percentage of proliferating cells within the AF connective tissue or osteogenic fronts of the paired frontal bones (*P*>0.02) (Fig. S3A-C). When these regions were combined, a statistically significant difference in proliferation of <1% was seen between control and *Wnt1-Cre;Fgfr2^−/−^* mice (6% in total control versus 5.1% in total mutant; *P*=0.02, *n*=7) (Fig. S3C). At P3, EdU pulse-chase showed a significant difference in proliferation of 2.3% between control and mutant mice (9.6% in total controls versus 7.3% in total mutant; *P*=0.004, *n*=6) (Fig. S3D-F). This difference was primarily driven by a decrease in the proliferative rate of the *Wnt1-Cre;Fgfr2^−/−^* AF connective tissue cells (6.8% in control versus 4.9% in mutants; *P*=0.001, *n*=6). Proliferation rates in the bone fronts were not statistically significantly different between mutant and control (Fig. S3F). Therefore, it is unlikely that a changes in proliferation in *Wnt1-Cre;Fgfr2^−/−^* mice lead to the persistent AF phenotype.

### Cells of the AF are non-autonomously regulated by *Fgfr2*

To determine the fate of AF cells during PFS formation, we traced *Scx-*lineage (Scx^LIN^) and *Sox9*-lineage (Sox9^LIN^) cells at P4 and P7. In *Scx-Cre;TdTomato:Ai9* mice at P4, Scx^LIN^ cells occupied the osteogenic fronts of the frontal bones, as well as the ecto- and endocranial layers of the AF (Fig. S4A). At P7, the contribution of Scx^LIN^ cells to the ecto- and endocranial layers of the AF had increased, occupying the mid-suture, osteogenic fronts of the frontal bones, and the suprasutural ligament (Fig. S4B). In *Sox9-CreERT2;TdTomato:Ai9* mice induced with tamoxifen at P2 and P3, Sox9^LIN^ cells were enriched in the endocranial domain of the AF and also contributed to the frontal bones at P4 (Fig. S4C). At P7, the Sox9^LIN^ cells contributed to the condensing cartilage in the endocranial domain of the PFS (Fig. S4D). These results show that different cell populations of the AF make direct and region-specific contributions to the PFS.

**Fig. 6.**
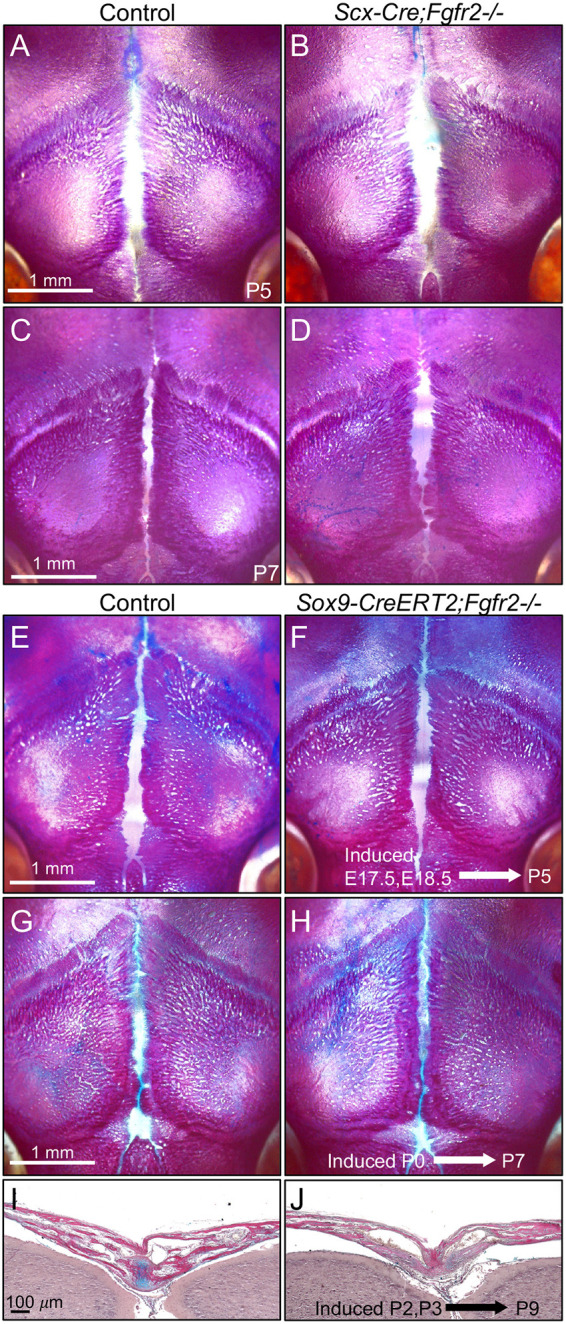
***Fgfr2* is not required in *Scx*^+^ or *Sox9*^+^ cells during AF closure.** (A-D) Wholemount Alizarin Red and Alcian Blue staining at P5 and P7 showed *Scx-Cre;Fgfr2^−/−^* mice, like their littermate controls, showed normal closure of the AF and formation of the PFS. *n*=3 littermate pairs per stage. (E,F) Wholemount Alizarin Red and Alcian Blue staining in *Sox9-Cre-ERT2;Fgfr2^−/−^* and littermate controls at P5 following tamoxifen induction at E17.5 and E18.5 showed normal AF closure. (G,H) Wholemount Alizarin Red and Alcian Blue staining in *Sox9-Cre-ERT2;Fgfr2^−/−^* and littermate controls at P7 following tamoxifen induction at P0 showed normal AF closure. (I,J) HBQ staining of the PFS at P9 following tamoxifen induction at P2 and P3 shows loss of cartilage (blue) in the endocranial layer in *Sox9-Cre-ERT2;Fgfr2^−/−^* mutants compared to littermate controls. *n*=4 littermate pairs per stage, per experiment.

We next tested the extent to which *Fgfr2* is required in *Scx*- and *Sox9*-expressing cells during AF development. Wholemount skeletal staining of *Scx-Cre;Fgfr2^−/−^* mice at P5 and P7 showed that the AF closure is indistinguishable from littermate controls (Fig. 6A-D). Since feature plots of the scRNA-seq dataset identified cells within the AF1 and AF2 clusters that co-express *Fgfr2* and *Scx* (Fig. S4E), we concluded that loss of *Fgfr2* in *Scx*^+^ cells was not sufficient to recapitulate the AF and PFS phenotypes seen in *Wnt1-Cre;Fgfr2^−/−^* mice. Wholemount skeletal staining of *Sox9-CreERT;Fgfr2^−/−^* mice at P5 following tamoxifen induction at E17.5 and 18.5 revealed that AF closure was indistinguishable from littermate controls (Fig. 6E,F). The same result was found when *Sox9-CreERT;Fgfr2^−/−^* mice were induced at P0 and collected at P7 (Fig. 6G,H). However, histological examination of the PFS in *Sox9-CreERT;Fgfr2^−/−^* mice at P9 following tamoxifen inductions at P2 and P3 showed loss of the endocranial cartilage (Fig. 6I,J). Feature plots of the scRNA-seq dataset identified cells within the AF2 and OF2 clusters that co-express *Fgfr2* and *Sox9* (Fig. S4F). Thus, although there is some autonomous requirement for *Fgfr2* during endochondral-like fusion of the PFS, it is not required in *Sox9*^+^ cells for AF closure.

### Fgfr2 regulates expression of WNT pathway members in the AF

**Fig. 7.**
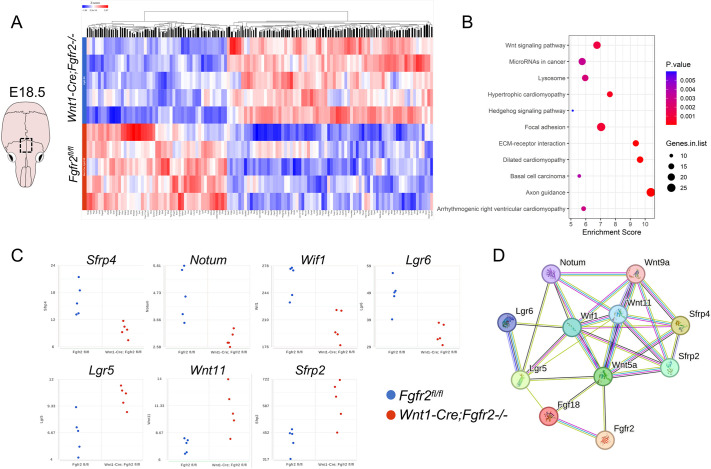
**Bulk RNA-seq reveals transcriptional differences in the AF of *Wnt1-Cre;Fgfr2^−/−^* embryos.** (A) Diagram of the E18.5 mouse calvarium showing the region (box) that was analyzed. Heatmap analysis revealing overall differences in gene expression between the AF of *Wnt1-Cre;Fgfr2^−/−^* (top rows) and littermate controls (bottom rows) (*n*=5 each for control and *Wnt1-Cre;Fgfr2^−/−^*). (B) GO analysis showing that differentially regulated genes were associated with focal adhesion, axon guidance, and WNT signaling. (C) Dot plots of differentially expressed genes associated with the WNT pathway showing that *Sfrp4*, *Notum*, *Wif1*, and *Lgr6* were downregulated in *Wnt1-Cre;Fgfr2^−/−^* mutants compared to controls. By contrast, *Lgr5*, *Wnt11*, and *Sfrp2* were upregulated in *Wnt1-Cre;Fgfr2^−/−^* mutants compared to controls. (D) String analysis inferred interactions between these differentially expressed WNT pathways members with *Fgfr2* and *Fgf18*. *n*=5 for control and Wnt1-Cre;Fgfr2^−/−^ littermate pairs.

To determine the molecular mechanisms through which *Fgfr2* regulates differentiation within the AF, we performed bulk RNA-seq of the control and *Wnt1-Cre;Fgfr2^−/−^* AF at E18.5, when a phenotypic difference was first detected. Heat map analysis of the differentially expressed genes showed that RNA expression profiles of control and *Wnt1-Cre;Fgfr2^−/−^* littermates have significant, genotype-specific differences (Fig. 7A). Gene ontology (GO) analysis of these genes indicated differential expression of members of the WNT signaling pathway, as well as genes related to focal adhesion, extracellular matrix–receptor interaction, and axon guidance (Fig. 7B). Previous studies have identified a role for WNT signaling in the developing suture, where this pathway works cooperatively with FGF signaling to determine skeletogenic cell fate (Liao et al., 2022; Quarto et al., 2010; Maruyama et al., 2010). Dot plots of WNT pathway members showed that secreted inhibitors *Sfrp4*, *Notum*, and *Wif1*, along with the WNT potentiator *Lgr6* were downregulated in the *Wnt1-Cre;Fgfr2^−/−^* AF (Fig. 7C). By contrast, the WNT/β-catenin target gene and WNT signaling potentiator *Lgr5*, *Wnt11*, and the context-dependent WNT modulator *Sfrp2* were upregulated in the *Wnt1-Cre;Fgfr2^−/−^* AF (Fig. 7C). String analysis of the WNT pathway genes found in our sequencing predicted likely interactions between the FGF and WNT pathways (Fig. 7D).

By comparing differentially expressed genes identified in the RNA-seq analysis with the cluster-enriched genes from the scRNA-seq dataset, we found that 40 of 42 AF1-enriched genes, 19 of 25 AF2-enriched genes, and 46 of 48 EM-enriched genes identified were upregulated in the *Wnt1-Cre;Fgfr2^−/−^* AF (Fig. S5). We found that 12 of 13 OF1-enriched genes, 16 of 16 OF2-enriched genes, and 57 of 57 OB-enriched genes identified were downregulated in the *Wnt1-Cre;Fgfr2^−/−^* AF. This suggests that loss of *Fgfr2* leads to increased expression of genes associated with AF connective tissue cell identity and decreased expression of gene associated with osteoblast identity.

**Fig. 8.**
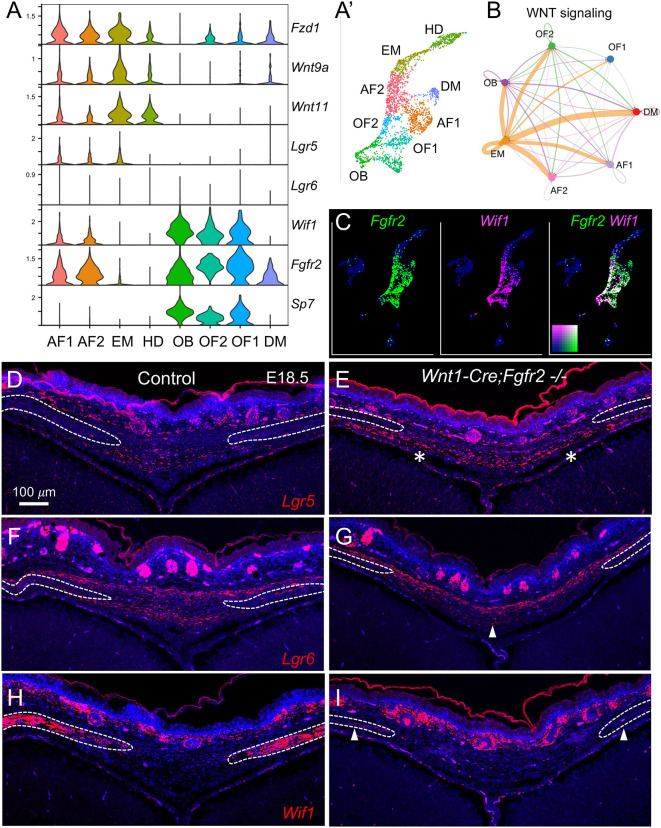
**Differentially expressed WNT pathway members correspond to distinct populations within the AF.** (A,A′) Violin plots (A) of WNT pathway members showing their cluster-specific enrichment in the developing AF at E18.5, with corresponding UMAP (A′). Expression of genes encoding the WNT receptor *Fzd1*, WNT ligands *Wnt9a* and *Wnt11*, and the WNT signaling target *Lgr5* were enriched in the AF1, AF2, and EM clusters, while expression of the WNT inhibitor *Wif1* was enriched in osteogenic populations OF1, OF2, and OB. (B) Cell communication analysis using CellChat predicted that EM is a crucial source of WNT ligand for AF1 and AF2. (C) Co-expression analysis of *Fgfr2* and *Wif1* showed significant overlap in OF1 and OF2 clusters (white cells). (D,E) RNAScope *in situ* hybridization in the AF at E18.5 identified low level expression of *Lgr5* throughout both ecto- and endocranial domains in the control. In the *Wnt1-Cre;Fgfr2^−/−^* mutant AF, the domain of *Lgr5* was expanded laterally towards the osteogenic fronts (asterisks). (F,G) *Lgr6* was expressed throughout the ecto- and endocranial domains of the AF in control, but expression within the endocranial domain was lost in the *Wnt1-Cre;Fgfr2^−/−^* mutant (arrowhead). (H,I) In control, *Wif1* was expressed throughout the osteogenic fronts. In the *Wnt1-Cre;Fgfr2^−/−^* mutant, *Wif1* expression was greatly decreased (arrowheads). Dashed lines delineate the frontal bones. *n*=3 littermate pairs.

Violin plots of WNT pathways members from the scRNA-seq dataset showed that the AF1, AF2, and EM clusters had enriched expression of the WNT receptor *Fzd1*, as well as *Wnt9a*, *Wnt11*, and *Lgr5* (Fig. 8A,A′). Cellular communication modeling using CellChat predicted that WNT ligands from the EM regulate WNT signaling in AF1 and AF2 (Fig. 8B). The OF1, OF2, and OB clusters, by contrast, were enriched for *Wif1* in cells that also expressed *Fgfr2* (Fig. 8A,A′,C). WNT-related gene expression changes identified through RNA-seq were validated using RNAScope *in situ* hybridization at E18.5. *Lgr5* expression, which was enriched in the endocranial domain of the control AF, was expanded in the *Wnt1-Cre;Fgfr2^−/−^* AF (Fig. 8D,E, asterisks). *Lgr6* expression, which was observed in both the endo- and ectocranial domains of the control AF, was reduced within the endocranial domain of the *Wnt1-Cre;Fgfr2^−/−^* AF (Fig. 8F,G, arrowhead). *Wif1* expression was localized to cells within the osteogenic fronts in the control and greatly reduced in *Wnt1-Cre;Fgfr2^−/−^* embryos (Fig. 8H,I, arrowheads). Together, these results indicate that *Fgfr2*-dependent activation of *Wif1* expression in cells of the osteogenic front non-autonomously modulates WNT signaling in *Lgr5-* and *Lgr6*-expressing cells of the AF. LGR5 is a known stem cell marker in multiple epithelial tissues, and in this context *Lgr5*-expressing cells may represent connective tissue progenitors (Leung et al., 2020; Ng et al., 2014; Shi et al., 2012).

### Modulation of WNT signaling impacts AF closure

**Fig. 9.**
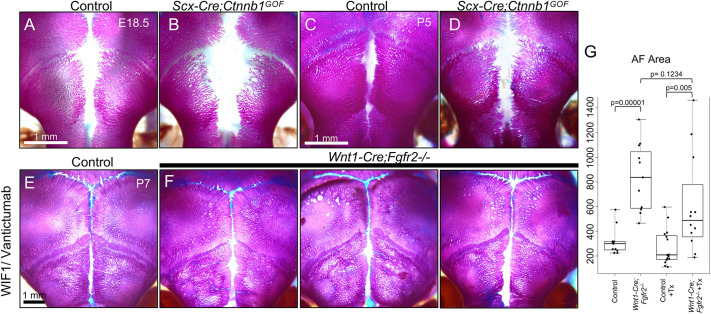
**Modulation of WNT signaling controls AF closure.** (A-D) Wholemount Alizarin Red and Alcian Blue staining at E18.5 and P5 showing that *Scx-Cre;Ctnnb1^GOF^* mice exhibit a wide-open AF that fails to close compared to littermate controls. *n*=3 littermate pairs per stage. (E-G) A WNT inhibitor cocktail composed of recombinant mouse WIF1 protein and vantictumab was administered at P3 through a single injection under the scalp in the area above the AF. (E) Wholemount Alizarin Red and Alcian Blue staining of a treated control collected at P7 showed that the treatment had no negative effects on calvarial bone formation and AF closure. (F) Wholemount skeletal stains of three treated *Wnt1-Cre;Fgfr2^−/−^* littermates showed that AF closure is partially restored. (G) Quantification of the AF area at P7 in control and *Wnt1-Cre;Fgfr2^−/−^* mice that were treated (Tx) with WIF1/vantictumab at P3 or untreated.

To test the impact of increased WNT/β-catenin signaling in *Scx*^+^ cells of the AF, we crossed mice harboring the *Scx-Cre* driver to mice with a stabilizing mutation in exon 3 of β-catenin [*Ctnnb1^lox(Ex3)^*] (Harada et al., 1999). Stabilization of the β-catenin protein blocks its degradation through the destruction complex and mimics conditions of constitutively active WNT signaling. Wholemount skeletal staining of *Scx-Cre;Ctnnb1^lox(Ex3)/+^* (*Scx-Cre;Ctnnb1^GOF^*) mice at E18.5 showed that the AF is larger compared to that of littermate controls (Fig. 9A,B). At P5, when the frontal bones of control mice approximate to form the PFS, the AF remained patent and the PFS failed to form in *Scx-Cre;Ctnnb1^GOF^* mice (Fig. 9C,D). The observation that increased WNT/β-catenin signaling in *Scx*^+^ cells recapitulate the phenotype of *Wnt1-Cre;Fgfr2^−/−^* mice supports a mechanistic connection between FGF and WNT signaling during AF closure and PFS formation.

To test the idea that elevated WNT signaling in *Wnt1-Cre;Fgfr2^−/−^* mice underlies the AF closure defect, we attempted a rescue through WNT signaling inhibition. To inhibit WNT signaling, we administrated a cocktail of mouse WIF1 recombinant protein and vantictumab, a humanized monoclonal antibody that inhibits WNT pathway signaling through competitive binding of FZD receptors. At P3, *Wnt1-Cre;Fgfr2^−/−^* pups and littermate controls were treated with a single injection of the WNT inhibitor cocktail just under the scalp at the location of the AF. Samples were collected at P7 and processed for wholemount skeletal staining. Following treatment, AF closure was largely restored in 9 out of 12 treated *Wnt1-Cre;Fgfr2^−/−^* mutants (Fig. 9E-G). Quantitative measurement showed that the median area of the AF was reduced in treated *Wnt1-Cre;Fgfr2^−/−^* (487.75; s.d.=398.49, *n*=12) versus untreated *Wnt1-Cre;Fgfr2^−/−^* mice (837; s.d.=275.43, *n*=11) (Fig. 9G). These results indicate that elevated WNT signaling is a crucial driver of the persistent AF phenotype in *Wnt1-Cre;Fgfr2^−/−^* mice.

## DISCUSSION

**Fig. 10.**
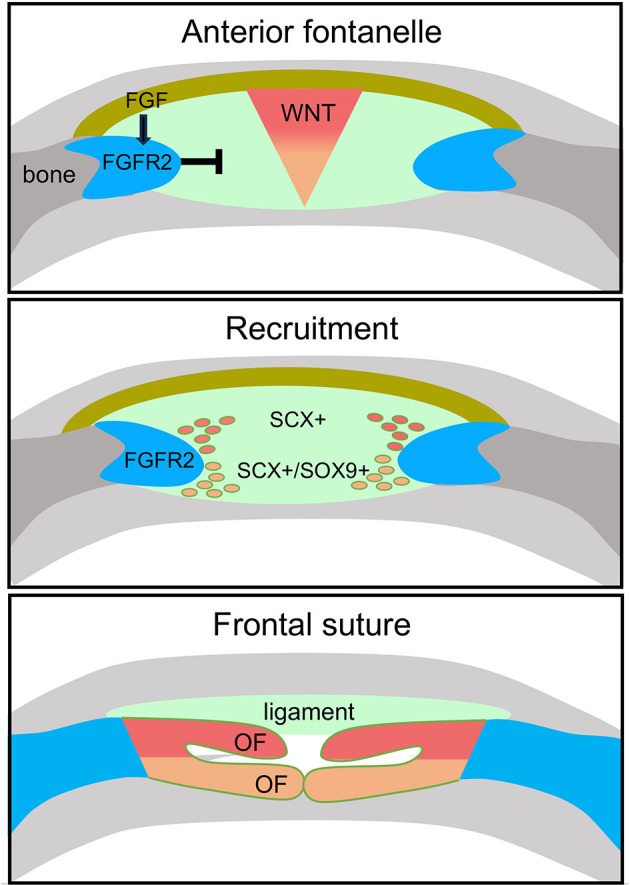
**Recruitment model for frontal suture formation within the AF.** A gradient of WNT signaling maintains cells of the AF in an undifferentiated state. As FGFR2^+^ cells of the osteogenic fronts advance, activated by FGF from the ectocranial mesenchyme, they secret WIF1 to locally inhibit WNT signaling in the AF. Downregulation of WNT signaling induces SCX^+^ cells in the ectocranial domain to form intramembranous bone, and SCX^+^/SOX9^+^ cells in the endocranial domain to form cartilage. Recruited SCX^+^ cells form the frontal suture, while SCX^+^ cells that remain outside the domain of WNT inhibition give rise to suprasutural ligament. Upon loss of FGFR2 signaling, WNT signaling remains high and AF cells fail to form the frontal suture joint.

This study characterizes a previously unappreciated role for FGF signaling in calvarial development. Using a combination of mouse genetics and single-cell transcriptomics, we demonstrate that *Fgfr2* is necessary for closure of the AF and development of the PFS it prefigures. We find that cells of the AF, which are marked by *Scx* and differentially express *Sox9*, are regionally organized into ecto- and endocranial domains that give rise to distinct lineages within the PFS. We provide evidence that FGFR2 signaling in the osteogenic fronts of the frontal bones, through induction of the expression of *Wif1*, induces local inhibition of WNT signaling in the AF (Fig. 10). Within the zone of WNT inhibition, SCX^+^ cells in the ectocranial domain are recruited to form bone, and SCX^+^/SOX9^+^ cells in the endocranial domain are recruited to form cartilage. A subset of ectocranial SCX^+^ cells that remain outside the domain of WNT inhibition give rise to suprasutural ligament. Upon loss of FGFR2 signaling, *Wif1* expression is lost, WNT signaling remains, and cells of the AF retain a connective tissue-like fate instead of forming the PFS.

Our results reveal that FGF and WNT pathways interact to coordinate frontal bone advancement with suture joint formation. We show that differential expression of FGF and WNT pathway members in cells of the ectocranial mesenchyme, osteogenic fronts, and AF establish a signaling center that promotes a unique lineage potential along the ectocranial-endocranial axis. Ectocranial mesenchymal cells express FGF and WNT ligands, suggesting that these cells are an essential source of secreted signals that act on *Fgfr2*^+^ cells in the osteogenic fronts and *Fgfr2*^+^ and *Fzd1*+ cells in the AF. FGF18 is likely the ligand responsible for promoting osteoblast cell fate in *Fgfr2*^+^ cells, as prior studies show that loss of *Fgf18* leads to wide-open fontanelles and ectopic FGF18 accelerates approximation of the bone fronts in the coronal suture (Ohbayashi et al., 2002; Sahar et al., 2005; Nagayama et al., 2013). WNT, by contrast, likely acts to maintain *Fzd1*^+^ cells in the AF in an undifferentiated state. This is supported by multiple studies showing that, in the PFS, canonical WNT signaling is dynamic and sharply downregulated immediately preceding cartilage formation in the endocranial domain while continuous activation of WNT leads to a wide-open frontal suture and failure of cartilage formation (Behr et al., 2010, 2011, 2013). We propose that *Fgfr2*^+^ cells at the osteogenic fronts downregulate WNT in the AF to promote chondrogenic fate through their expression of the secreted WNT inhibitor WIF1. That WNT promotes connective tissue over cartilage fate is also supported by studies in the developing limb (ten Berge et al., 2008).

The calvaria develops from a layer of head mesenchyme that is derived from mesoderm or neural crest, and regionally patterned into osteogenic and non-osteogenic domains. Mesenchyme that initially resides in the supraorbital ridge just above the eyes, called the supraorbital mesenchyme (SOM), generates the ossification centers for the frontal and parietal bones (Ishii et al., 2015; Deckelbaum et al., 2012; Jiang et al., 2002; Yoshida et al., 2008). In contrast, mesenchyme that lies apical to the SOM, called the early migrating mesenchyme (EMM), remains non-osteogenic through embryonic stages of calvarial development (Cesario et al., 2018; Roybal et al., 2010). Growth of the frontal and parietal bone rudiments towards the vertex of the head in embryogenesis is fueled by the SOM through intrinsic growth rather than recruitment of adjacent EMM mesenchyme (Yoshida et al., 2008). Instead, the EMM contributes to non-osteogenic connective tissue that occupies the sutures and fontanelles (Cesario et al., 2018). Our findings elaborate on this model by showing that, in the postnatal period, non-osteogenic SCX^+^ cells within the AF directly contribute to bone, cartilage, and suprasutural ligament within the PFS. This suggests that formation of the PFS within the AF is developmentally distinct. Our results indicate that, as the frontal bones approximate, they recruit SCX^+^ cells of the AF to initiate frontal suture joint formation, which is characterized by the formation of a secondary osteogenic front within the endocranial layer that subsequently undergoes endochondral fusion. This may be a key factor that distinguishes the bilayered, fusing murine PFS from the overlapping coronal suture or abutting sagittal suture, which do not fuse during normal mouse development. Importantly, SCX^+^ cells in the AF appear to be distinct from those we previously identified in the non-fusing murine coronal suture. In the coronal suture, SCX^+^ cells are highly restricted to the overlapping edges of the frontal and parietal bones in the ectocranial domain and appear to remain non-osteogenic (Farmer et al., 2021).

Overall, the results presented in this study offer further insight into regional differences in the development of sutures that are prefigured by fontanelles. How might the mechanism of AF closure identified here explain why both an increase and decrease in FGFR2 signaling lead to failed formation of sutures that develop within fontanelles and why an increase in FGFR2 signaling causes AF patency along with premature fusion of other cranial sutures? Although it is not yet clear how *FGFR2* gain-of function variants impact the mechanism for AF closure we described here, our study indicates that suture-specific requirements for FGF and/or WNT signaling levels could be the key to answering these questions. One possibility is that the AF phenotype observed in a model of FGFR2 loss of function leads to excess unbound ligand that can diffuse and activate signaling in regions where FGF signaling is normally low, recapitulating a gain of function via other receptors. Specifically, whereas FGFR2 is expressed in the osteogenic fronts and AF near a source of FGF ligands, FGFR1 and FGFR3 are expressed in osteoblasts located in an adjacent domain (Johnson et al., 2000; Iseki et al., 1999; Rice et al., 2000). Another possibility is that an increase or decrease in FGFR2 signaling leads to WNT activation through distinct mechanisms. Interestingly, in a mouse model for Apert syndrome, which harbors the gain-of-function variant *Fgfr2^S252W^* that causes both craniosynostosis and a persistent AF, the premature fusion of the coronal suture has been linked to activation of WNT signaling through increased expression of the Frizzled co-receptors *Lrp5* and *Lrp6* (Min Swe et al., 2020). Increased WNT signaling may also underlie persistent AF patency in other craniosynostosis syndromes. A previous study found that *Lgr5* is upregulated in the developing frontal suture of *Twist^+/−^* mice, a model for Saethre–Chotzen syndrome, which presents with persistent AF and premature coronal suture fusion (Holmes et al., 2020).

## MATERIALS AND METHODS

### Mice

To conditionally knockout *Fgfr2* in neural crest cell (NCC)-derived tissue, *Fgfr2^flx/flx^* (The Jackson Laboratory, stock #007569) mice were crossed with the *Wnt1-Cre* driver (The Jackson Laboratory, stock #003829), the *Sox9-CreERT2* driver (The Jackson Laboratory, stock #035092) (Akiyama et al., 2005), or the *Scx-Cre* driver, which was generously provided by Dr Ronen Schweitzer (Shriners Hospital for Children, Oregon Health and Science University; Portland, OR, USA). The *Fgfr2^flx/flx^* and *Wnt1-Cre* lines were previously described (Lewis et al., 2013; Yu et al., 2003). *Scx-GFP* was used to mark dense connective tissue fibroblasts (Pryce et al., 2007). *Scx-Cre* and *Sox9-Cre-ERT2* lines were described previously (Xu et al., 2015; Sugimoto et al., 2013). The *Ai9* allele (The Jackson Laboratory, stock #007909) was used as a lineage marker for those tissues targeted by *Scx-Cre* and *Sox9-Cre-ERT2* (Madisen et al., 2010). For *Sox9-Cre-ERT2* mice, tamoxifen (Sigma-Aldrich; dissolved in peanut oil overnight at 40°C) was delivered via oral gavage to either timed pregnant females (for embryonic inductions; 1 mg/induction/dam) or neonatal mice (for postnatal inductions; 0.1 mg/induction/pup). Finally, constitutive activation of β-catenin in fibroblasts was driven by crossing *Ctnnb^lox(Ex3)^* mice (Harada et al., 1999) to mice carrying the *Scx-Cre* driver. Embryonic samples were collected from timed pregnant females. Postnatal samples were staged according to the date of birth. All experimental protocols were approved by the USC's Institutional Animal Care and Use Committee.

### Skeletal preparation

Mice processed for wholemount imaging were collected as above and following a PBS rinse were fixed in 95% ethanol for a minimum of 4 days. Fixed samples were stained for cartilage using 0.15 mg/ml Alcian Blue (Sigma-Aldrich, A5268) in 80% ethanol and 20% glacial acetic acid. Samples were then de-stained in 95% ethanol for a minimum of 2 days. Tissue clearing was performed using KOH between 0.5 and 1% w/v for 2-8 days depending on the size of the sample. Calcified tissue was then discriminated using 0.02 mg/ml Alizarin Red S (Sigma-Aldrich, A5533) in 0.5-1% KOH. Destaining and further clearing was performed as needed in 0.5-1% KOH. Samples were equilibrated, stored, and imaged in 75% glycerol. Measurements of the AF area using ImageJ and each individual area measurement were normalized to the length of the PFS from the jugum limitans to the coronal suture. These were then averaged within each group and the average AF area of control and *Wnt1-Cre;Fgfr2^−/−^* mice was compared by age using an unpaired, two-tailed *t*-test assuming unequal variance.

### μCT

All μCT scans were performed by the USC Molecular Imaging Center using a μCT50 (Scanco Medical). Samples were rotated 360° and X-ray settings were standardized to 90 kV and 155 µA with an exposure time of 0.5 s per frame to yield a nominal resolution of 20 μm. A 0.5-mm-thick aluminum filter was employed to minimize beam-hardening artifacts. Morphometric analysis was performed using Amira 6.2 and VG Studio MAX 3.0 software packages. Isosurface renderings with equal threshold were measured using the 3D measuring tool. All jaw measurements and landmarks were measured as previously described (Guerreiro et al., 2013). These experiments were performed on at least three biological replicates, which we defined as three same-sex littermate pairs (control and mutant) derived from three different litters. Statistical significance was determined using unpaired, two-tailed Student's *t*-tests.

### Histology

Samples used for histology were processed as follows. Animals were euthanized humanely and decapitated. Heads were then fully or partially skinned, rinsed in PBS, and fixed in 10% neutral buffered formalin at room temperature for 24-36 h depending on stage. Following fixation, samples were decalcified in 10% EDTA (pH 7.4) at 4°C for 3-7 days, until calvaria began to concave. Samples were then dehydrated in serial ethanol washes of increasing concentration, equilibrated in Citrus Clearing Solvent (VWR, 72060-044), and embedded following three washes in pure paraffin wax at 65°C with vacuum pressure. Embedded samples were then cut into 8-µm-thick sections. Sections were stained using the Hall-Brunt quadruple stain (Hall, 1986).

### Immunofluorescent analysis

Samples for immunofluorescent staining were harvested and fixed in 4% paraformaldehyde at 4°C for 30-90 min depending on stage. Decalcification of bone was performed by incubation in 10% EDTA (pH 7.4) at 4°C for 3-7 days until calvaria began to concave. Subsequently, samples were equilibrated sequentially in 15% and 30% sucrose/PBS at 4°C until they sank, then embedded in optimal cutting temperature (O.C.T.) compound (Electron Microscopy Sciences). Cryosectioning was performed at a section thickness of 8 μm onto SuperFrost Plus slides. Sections were washed three times with PBS, permeabilized with 0.1% Triton X-100 in PBS, then blocked for 1 h at room temperature in 10% serum (either donkey or goat from Sigma-Aldrich). Slides were incubated in primary antibody overnight at 4°C (Table S1), then washed in PBS and incubated in Alexa Fluor 568 secondary antibody (Thermo Fisher Scientific A-11036) at a concentration of 1:200 for 1 h at room temperature. Finally, slides were washed in PBS and mounted in VECTASHIELD with DAPI (Vector Laboratories). Slides used for lineage tracing were washed three times in PBS and counterstained with DAPI as described above. Slides were imaged using either a Leica TCS SP5/8 or Stellaris 5 confocal system. These experiments were performed on three biological replicates, which we defined as three littermate pairs (control and mutant) derived from two different litters.

### Single-cell transcriptomics

Data for scRNA-seq analysis was obtained from FaceBase (accession 1-4TT6) (Holmes et al., 2020). Seurat v.3 R-Package was used to explore QC metrics and filter for high-quality cells (Stuart et al., 2019). The filtered dataset representing 3366 cells (median of 3750 genes per cell) was normalized (log normalized), scaled (linear transformation), and clustered into 16 clusters (dims 1:13, resolution=0.7) using unsupervised graph-based clustering based on principal component analysis scores. The identities of the clusters were resolved using previously reported markers. Pseudotime analysis was conducted using Monocle 3 (Cao et al., 2019). Cell–cell communication predictions were made using CellChat (Jin et al., 2021).

### *In situ* hybridization

Samples for *in situ* hybridization were collected and processed in the same manner as histological samples, described above. Transcripts were detected using RNAScope Fluorescent Multiplex Assay v2 (ACD) as per manufacturer's instructions. Briefly, slides with paraffin sections were baked in an embedding oven for 60 min at 65°C. Slides were then deparaffinized using xylenes, dehydrated in 100% ethanol, and allowed to completely air dry. Endogenous peroxidase activity was quenched using hydrogen peroxide provided within the kit and slides were washed in deionized water. Antigen retrieval was performed using the provided Target Retrieval Reagent for 15 min in an Oster steamer heated to 99°C. Slides were then dehydrated in 100% ethanol and allowed to completely air dry. The slides were treated with ACD Protease plus in a humidified slide chamber for 15 min at 40°C. Probe hybridization was also performed in a 40°C humidified slide chamber for 2 h (Table S1). Slides were then stored in 5× SSC solution overnight. Amplification steps were performed as prescribed by the manufacturer and signal development was carried out using Opal 570 (Akoya Biosciences, FP1488001KT) Opal 620 (Akoya Biosciences, FP1495001KT) or Opal 690 (Akoya Biosciences, FP1497001KT) diluted 1:1000 in the ACD provided TSA buffer. Slides were then counterstained using VECTASHIELD mounting medium with DAPI and imaged using the Leica TCS SP5/8 or Stellaris 5 confocal system. These experiments were performed on three biological replicates, which we defined as three littermate pairs (control and mutant) derived from two different litters.

### EdU proliferation assay

Cell proliferation was assayed using an *in vivo* EdU Click kit according to the manufacturer's instructions (Sigma-Aldrich, BCK647-IV-IM-M). Briefly, E18.5 samples were obtained by intraperitoneally injecting timed pregnant females with 1.5 mg EdU dissolved in PBS, and P3 mice received subcutaneous injections of 0.1 mg EdU dissolved in PBS. In both cases, a chase period of 4 h was observed before sample collection. Samples were cryo-embedded and sectioned at 8 μm as described above. Detection of EdU took place in the dark, at room temperature for 30 min. Slides were then counterstained and mounted using VECTASHIELD mounting media with DAPI. For analysis, littermate control and *Wnt1-Cre;Fgfr2^−/−^* pairs were selected, and a minimum of six different sections from each sample were imaged and uploaded to ImageJ. Each image was quantified for total AF cells by counting individual nuclei, and the number of proliferating cells was counted. Statistical analysis was performed on total proliferation in mutants compared with controls, proliferation of AF fibrous connective tissue, and proliferation in bone front cells. Statistical significance was determined using unpaired, two-tailed *t*-tests assuming unequal variance.

### RNA isolation and gene expression analysis

The region of the AF was excised from E18.5 embryos (including both suture connective tissue and bone but excluding skin and brain) and added to 300 µl of DNA/RNA Shield (Zymo Research). RNA was isolated using the Quick-RNA Miniprep Plus Kit (Zymo Research) according to the manufacturer's instructions with the addition of an optional DNase digestion and a 1 min dounce homogenization step following proteinase-K digestion. Samples were eluted from the column using 80 µl of DNase/RNase-free water and then analyzed using a NanoDrop spectrophotometer and to check concentration and purity. Library preparation and sequencing was performed at the UCLA TCBG facility using the Kapa Stranded Kit (Roche). Sequencing was performed using HiSeq 3000 at 1×50 read length and 30 million reads per sample. Differential gene expression analysis was performed using Partek Flow.

### WNT inhibitor cocktail treatment

Stock vantictumab (Fisher Scientific, MA5-42006) and recombinant WIF1 protein (R&D Systems, 135-WF-050) were diluted in sterile water to a final concentration of 1.1 mg/ml and 0.5 mg/ml, respectively. At P3, pups were treated with a single dose of the inhibitor cocktail (a total of 2 μg vantictumab and 2 μg Wif1) using a pre-filled 29 G insulin syringe. For the treatment, pups were placed on a nitrile glove on ice for 30-45 s to slow movement, and the WNT inhibitor cocktail was injected subcutaneously just under the scalp above the area of the AF. Volume of liquid per injection did not exceed 20 μl. Following treatment, pups were placed on a heating pad and observed for several minutes to ensure recovery from the procedure before returning to their dams.

## Supplementary Material



10.1242/develop.204264_sup1 Supplementary information
