# Supplementary material for: FGFR2 directs inhibition of WNT signaling to regulate anterior fontanelle closure during skull development
Source: Development. 2025 Jan 20;152(2):dev204264. doi: 10.1242/dev.204264 (PMC11829768; doi:10.1242/dev.204264)
Supplement: Supplementary information [file develop-152-204264-s1.pdf]

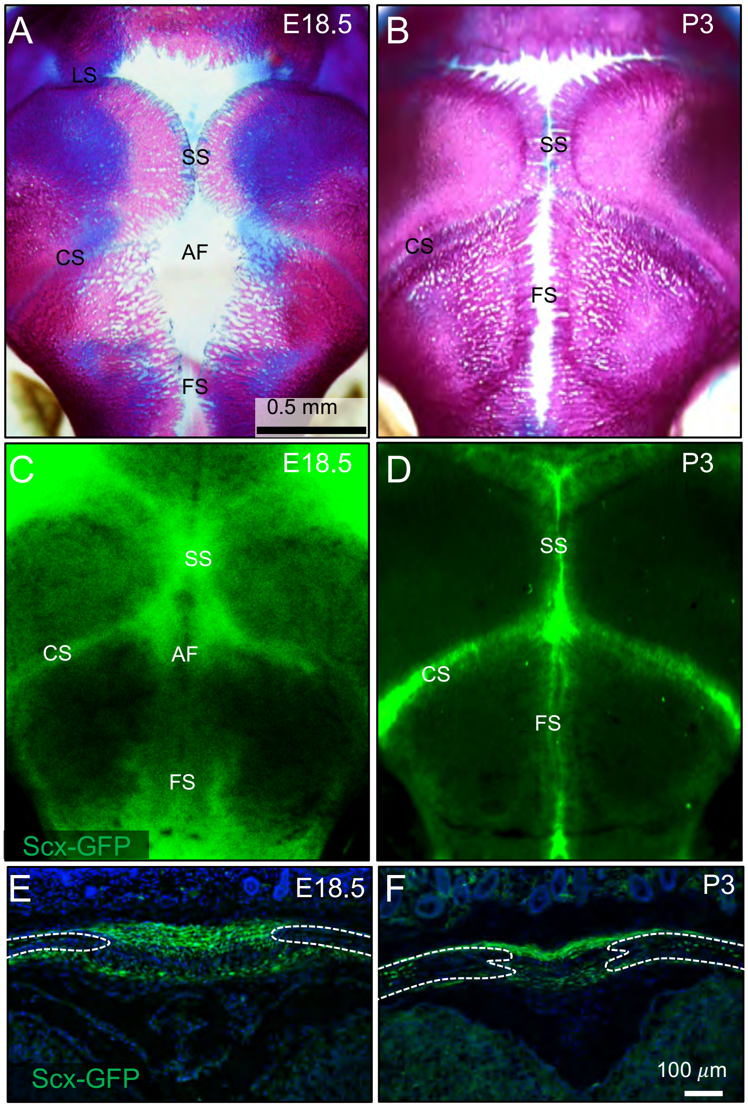

Fig. S1. Scx-GFP, a reporter for tendon/ligament, is expressed in the AF and PFS. (A,B) Wholemount alizarin red and alcian blue staining of the calvaria at E18.5 and P3 was used to delineate the calvarial bones. (C) At E18.5, Scx-GFP was expressed throughout the connective tissue of AF, as well as in the coronal and sagittal sutures. (D) At P3, when the AF is being replaced by the PFS, Scx-GFP expression was more confined to the sutures themselves. (E,F) Coronal sections through the anterior fontanelle at E18.5 and P3 showed a bi-layered expressional pattern of Scx-GFP, representing the ecto- and endocranial layers. Osteogenic fronts are outlined with dotted white lines. AF, anterior fontanelle; CS, coronal suture; FS, frontal suture; SS, sagittal suture.

Development • Supplementary information

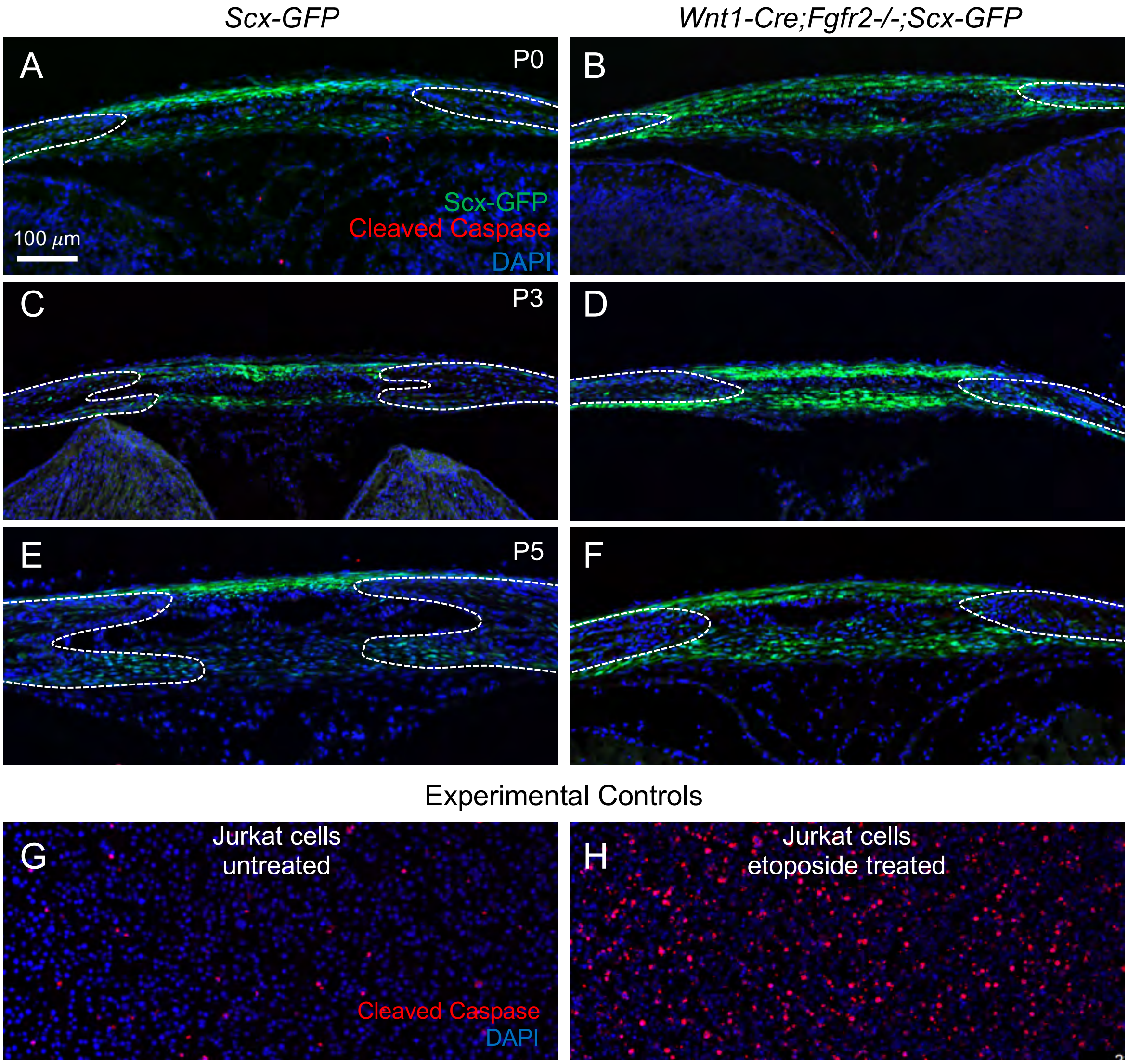

Fig. S2. Cleaved caspase staining shows no significant levels of apoptosis in the developing AF of control or *Wnt1-Cre;Fgfr2$^{-/-}$* mice. Immunofluorescence staining of cleaved caspase at P0 (A,B), P3 (C,D), and P5 (E,F) showed no significant levels of apoptosis in either *Wnt1-Cre;Fgfr2$^{-/-}$* or littermate control mice (n=3 pairs for each stage). Antibody viability was confirmed with etoposide treated and untreated control slides (CST 8104S) (G,H).

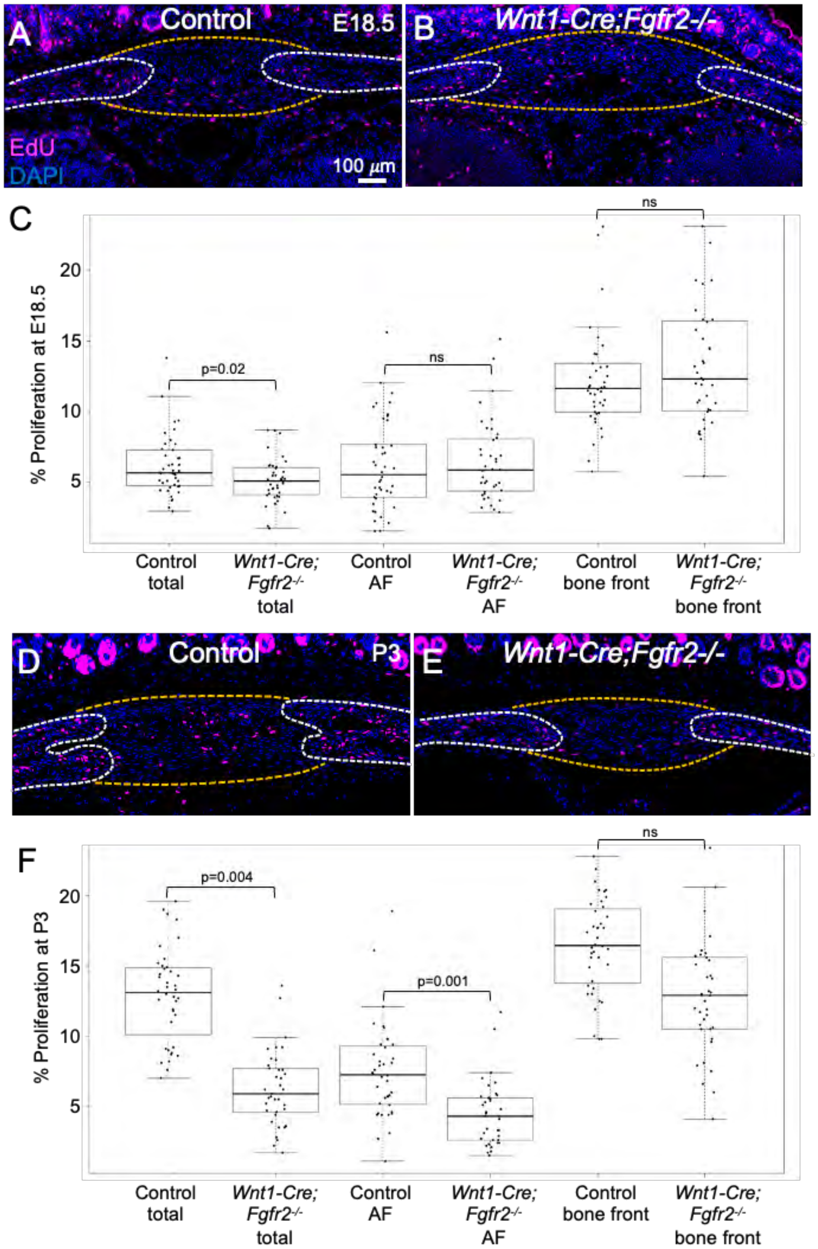

Fig. S3. EdU pulse chase identifies a minor decrease in proliferation in the AF of Wnt1-Cre; *Fgfr2*$^{-/-}$ mutant mice. (A-C) EdU pulse chase at E18.5 in control (A,C) and *Wnt1-Cre;Fgfr2*$^{-/-}$ littermates (B,C) identified a similar average percent of EdU+ (pink) proliferating cells within the osteogenic bone fronts (white dotted lines) and AF (yellow dotted lines). A significant difference in proliferation was only seen when counting both the bone front and AF regions (6% in control vs. 5.1% in mutant, p=.02) (n = 7 littermate pairs). (D-F) At P3, total proliferation was elevated in control samples (9.6% in control vs. 7.3% in mutant, p= .004) with the bulk of this difference occurring in the AF connective tissue (6.8% in control vs. 4.9% in cKO, p= .001) (n = 6 littermate pairs). Statistical significance was calculated using t-test assuming unequal variance.

Development • Supplementary information

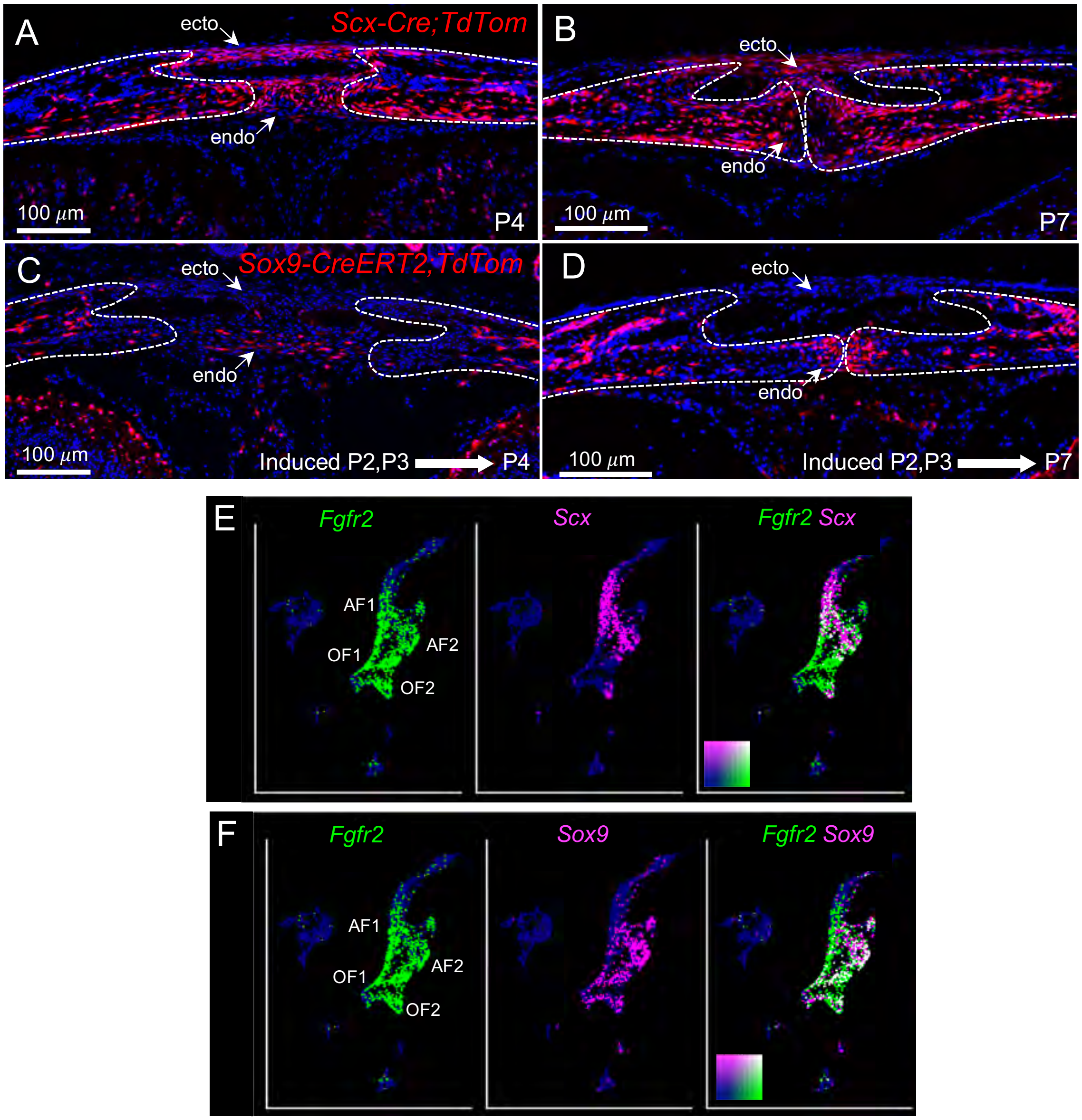

Fig. S4. *Scx*-lineage and *Sox9*-lineage cells directly contribute to the developing PFS. (A) Genetic lineage tracing in *Scx-Cre;TdTomato:Ai9* mice showed widespread contribution of Scx^{LIN} cells at P4 and P7 to both the osteogenic fronts of the frontal bones and the AF connective tissue. (B) At P7, when the PFS has been established, Scx^{LIN} cells contributed to the ecto- and endocranial bone, as well as the suprasutural ligament. (C) Genetic lineage tracing in *Sox9-CreERT2;TdTomato:Ai9* mice following tamoxifen induction at P2 and P3, identified Sox9^{LIN} in the osteogenic fronts of the frontal bones and within the endocranial domain of the AF at P4. (D) At P7, the Sox9^{LIN} cells contributed to the cartilage condensation within the endocranial domain. Dotted lines in each panel outline osteogenic fronts. (N = 3 per stage). (E) Co-expression analysis in the scRNA-seq data indicated that *Scx*+ cells within the AF1 and AF2 clusters express *Fgfr2* at E18.5. Individual cells are color coded as either *Fgfr2*+ only (green), *Scx*+ only (pink), *Fgfr2+/Scx+* simultaneously (white) or *Fgfr2-/Scx-* (blue). (F) Co-expression analysis in the scRNA-seq data showed *Sox9*+ cells within the AF2 and OF2 clusters express *Fgfr2* at E18.5. Individual cells are color coded in the same manner as in E.

Development • Supplementary information

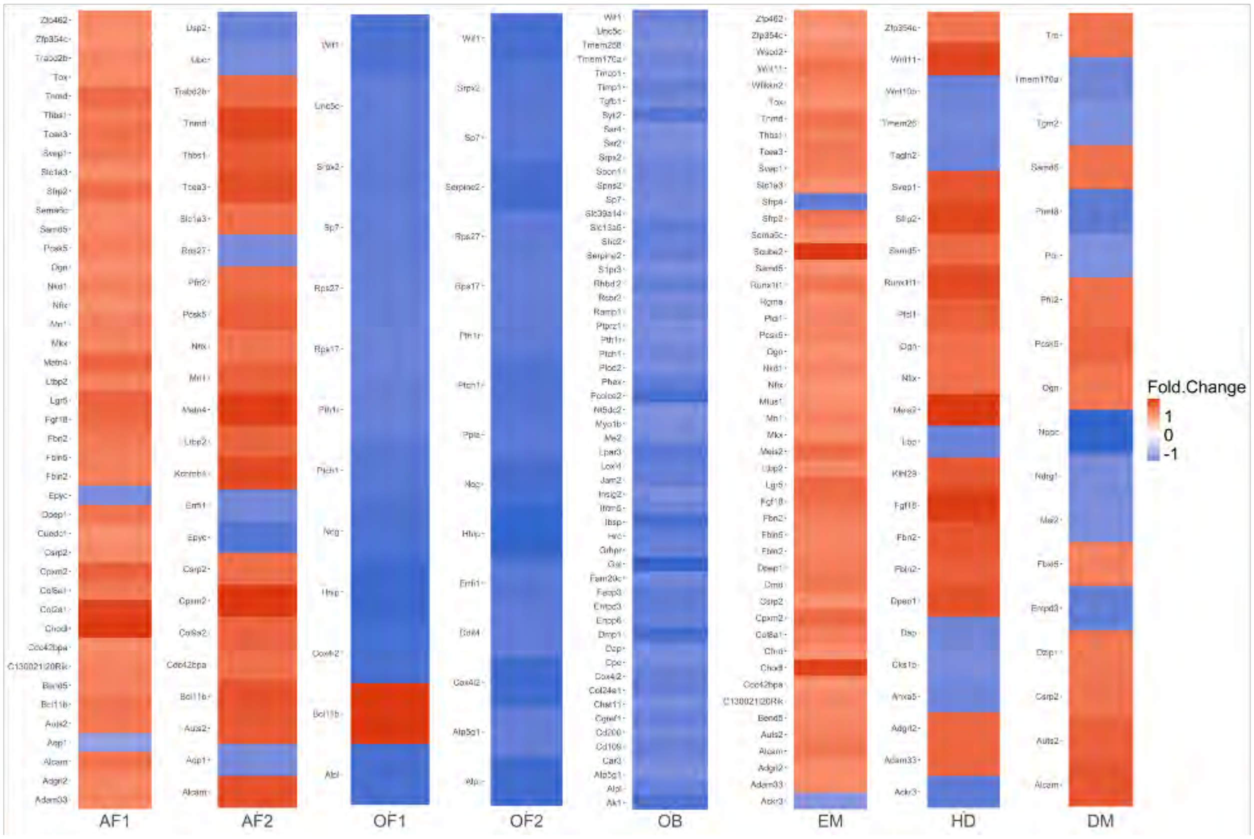

Fig. S5. Gene associated with AF identity are upregulated, while those associated with osteogenic identity are downregulated in the AF of Wnt1-Cre; *Fgfr2*$^{-/-}$ mutant embryos at E18.5. Differentially expressed genes identified in the bulk RNA-seq analysis were compared to cluster-enriched genes identified in the scRNA-seq dataset to reveal cluster-specific trends. Heat maps show cluster-enriched genes that were either up- or down-regulated in the *Wnt1-Cre;Fgfr2*$^{-/-}$ AF. We found that 40 of 42 AF1-enriched genes, 19 of 25 AF2-enriched genes, and 46 of 48 EM-enriched genes identified were upregulated in the *Wnt1-Cre;Fgfr2*$^{-/-}$ AF. On the other hand, we found that 12 of 13 OF1-enriched genes, 16 of 16 OF2-enriched genes, and 57 of 57 OB-enriched genes were downregulated in the *Wnt1-Cre;Fgfr2*$^{-/-}$ AF.

Development • Supplementary information

Table S1. List of in situ probes and antibodies used in the study.

Target	Catalog #	Application	Concentration
Fgf18	ACD 495421	RNAscope *in situ*	1:50
Fgfr2	ACD 443501-C2	RNAscope *in situ*	1:50
Lgr5	ACD 312171-C2	RNAscope *in situ*	1:50
Lgr6	ACD 404961	RNAscope *in situ*	1:50
Scx	ACD 439981	RNAscope *in situ*	1:50
Sox9	ACD 401051	RNAscope *in situ*	1:50
Sp7	ACD 403401-C3	RNAscope *in situ*	1:50
Tnmd	ACD 430531	RNAscope *in situ*	1:50
Wif1	ACD 412361-C3	RNAscope *in situ*	1:50
Opal 570	Akoya Biosciences FP1488001KT	RNAscope *in situ*	1:1000
Opal 620	Akoya Biosciences FP1495001KT	RNAscope *in situ*	1:1000
Opal 690	Akoya Biosciences FP1497001KT	RNAscope *in situ*	1:1000
Cleaved Caspase 3 (rabbit)	CST 9661	Immunofluorescence	1:400
Runx2 (rabbit)	CST 12556	Immunofluorescence	1:400
Sox9 (rabbit)	Novus NBP1-85551	Immunofluorescence	1:400
Anti-Rabbit IgG Alexa Fluor 568 (goat)	ThermoFisher Scientific A-11036	Immunofluorescence	1:200
