# Supplementary material for: FGFR2 directs inhibition of WNT signaling to regulate anterior fontanelle closure during skull development
Source: Development. 2025 Jan 20;152(2):dev204264. doi: 10.1242/dev.204264 (PMC11829768; doi:10.1242/dev.204264)
Supplement: Supplementary file 1 [file develop-152-204264_review_history.pdf]

Development

FGFR2 directs inhibition of WNT signaling to regulate anterior fontanelle closure during skull development

Lauren Bobzin, Audrey Nickle, Sebastian Ko, Michaela Ince, Aaron Huang, Arshia Bhojwani, Ryan Roberts and Amy E. Merrill

DOI: 10.1242/dev.204264

Editor: Anna-Katerina Hadjantonakis

Review timeline

Original submission:	15 July 2024
Editorial decision:	23 August 2024
First revision received:	18 November 2024
Accepted:	17 December 2024

Original submission

First decision letter

MS ID#: dev.204264

MS TITLE: FGF Signaling Regulates Development of the Anterior Fontanelle

AUTHORS: Lauren Bobzin, Audrey Nickle, Sebastian Ko, Michaela Ince, Aaron Huang, Arshia Bhojwani, and Amy E. Merrill

I have now received all the referees reports on the above manuscript, and have reached a decision. The referees' comments are appended below, or you can access them online: please go to .

The overall evaluation is positive and we would like to publish a revised manuscript in Development, provided that the referees' comments can be satisfactorily addressed. Please attend to all of the reviewers' comments in your revised manuscript and detail them in your point-by-point response. If you do not agree with any of their criticisms or suggestions explain clearly why this is so. If it would be helpful, you are welcome to contact us to discuss your revision in greater detail. Please send us a point-by-point response indicating your plans for addressing the referees' comments, and we will look over this and provide further guidance.

Reviewer 1

Advance summary and potential significance to field

In this manuscript from Bobzin et al., the authors perform a histological and gene expression characterization of anterior fontanelle (AF) closure phenotypes that are observed upon ablation of the FGFR2 from neural crest in conditional mouse models. New analysis of existing scRNA-seq datasets provides deeper understanding of the organization of distinct cell types within the AF. Marker analysis and reporter mouse lines reveal impacts on osteogenic differentiation and a complete loss of cartilage differentiation within the AF, but a relatively minimal decrease in cell proliferation. Bulk RNA-sequencing indicates that WNT signaling is impacted by FGFR2 loss by virtue of changes in expression of secreted WNT inhibitors within FGFR2-expressing populations; further genetic and pharmacologic analysis in mice demonstrates the involvement of WNT signaling in AF

© 2024. Published by The Company of Biologists under the terms of the Creative Commons Attribution License (https://creativecommons.org/licenses/by/4.0/).

closure and supports the hypothesis that FGFR2 acts at least in part through modulation of WNT signaling. Overall, this is a very well-written manuscript with visually beautiful and convincing data. In most cases (see below), assertions are well supported by the data provided, and the overall conclusions are sound. These findings will be of significant interest to those who study calvarial/craniofacial skeletal development and are of particular relevance to human skull vault malformation conditions in which FGFR signaling is disrupted. Several minor points might be addressed to strengthen the manuscript.

Comments for the author

* In several cases, the authors might consider additional arrows to point out specific structures:
o The "cartilaginous fusions that follow a joint-gap pattern" in controls in Figure 1 I, should be indicated with an arrow or similar.
o Figure 2 might benefit from arrows that point to ectocranial and endocranial domains, secondary osteogenic fronts, and bone fronts in general, as the bone front is not obvious without careful examination.
o In Figure 6A-F, it might be helpful to mark with arrows the SCX and SCX/RUNX2 cells that are being referred to in the text. In this figure, higher magnification views of the bone fronts would also be helpful.
o In Figure 8C and D the distinct Sox9 lineage cells that are being described in the text (endocranial AF/frontal bone, and endocranial domain of the PFS) might be highlighted with arrows for readers.

* There is a typo on line 132; I believe it should read "posterior frontal".
* Typo: on line 174 "indicates that" is duplicated.

* On line 186, the authors indicate that "loss of Fgfr2 leads to a reduction of cells with the AF2 and OF2 identity. This seems potentially overstated, as this finding is based only on the expression of very few markers, which might be altered while cells remain present. The ideal data would be scRNA-seq on Fgfr2 mutants, but that would be quite a lot of work- can bulk RNA-seq data be used to further support this conclusion?

* At line 745, the authors suggest that "a small decrease in the proliferation rate of AF connective tissue could contribute to AF patency" But it is not clear to me how a decrease in proliferation in the AF population fits with the observation of expanded AF- if proliferation contributed, wouldn't it be expected that these cells would have increased proliferation? Clarification would be helpful.

* While the rescue of Fgfr2 loss with Wif1 injection appears impressive, rescue experiments in Figure 14 should include some sort of quantitative and statistical analysis to reflect the extent and consistency of rescue across the n=7 replicates compared with mock injection controls.

* In the discussion, the authors suggest that a similar etiology may exist for persistent AF patency in FGFR2 GOF, but it is not clear what is meant by this. Obviously, examination of AF phenotypes in GOF models is beyond the scope of this study, but further explanation of what is meant, and how these apparently opposite impacts on FGFR signaling could possibly result in the same AF phenotype is warranted in general, further discussion comparing the findings of this study with those that have investigated mechanisms of suture closure in Fgfr GOF synostosis models (e.g. is WNT signaling known to be impacted in those models?) would be of interest to better understand the scope and context of the mechanism that is uncovered here.

<u>Reviewer 2</u>

Advance summary and potential significance to field

The manuscript by Bobzin et al., addresses the question regarding the role of Fgfr2 in determining fate of various cell populations that contribute to calvarial sutures. Using various transgenic lines

© 2024. Published by The Company of Biologists under the terms of the Creative Commons Attribution License (https://creativecommons.org/licenses/by/4.0/).

and transcripional profiling, the authors conclude that FGFR2, within the osteogenic fronts, inhibits Wnt signaling within the anterior fontanelle. In the absence of Wnt activity, Scx+ cells are able to differentiate into bone, whereas Scx+/Sox9+ cells differentiate into cartilage. Loss of FGFR2 allows for increased Wnt activity and prevents progenitor differentiation into bone and cartilage of the suture. These findings improve our understanding of variation of cell populations within the developing suture and further explain the role of Fgf and Wnt signaling in this area. Overall this manuscript has a clear focus, high quality data, and logical conclusions and is likely to be of interest to those in the field.

Comments for the author

Major suggestions:
1. The various levels of expression of FgfR2 in different cell populations is interesting and begs the question of thresholds of Fgf activity (e.g., high in OF1/2 but low in AF1/2). Is there any evidence of 'high' or 'low' FGF activity priming cells for different fate decisions, as is the case for Wnt? If so this may be a topic for discussion.
2. Which isoform of Fgfr2 is being expressed in these cell populations? Is it the same isoform in all populations? Given the variability and concordant functional implications, it might be worthwhile to explore isoform expression.
3. Is Fgf18 the only ligand in the area? Some conclusions may be overstated regarding Fgfr2-Fgf18 if other ligands are expressed.
4. Is the repression of Wnt direct? If so what is the mechanism?
5. Has the lack of expression of Fgfr2 been confirmed through RNAscope in the NCC-Fgfr2-/- samples?

Minor:
1. The current title seems broad and does not highlight the uncovered mechanism. Consider revising to incorporate the Wnt dependent mechanism.
2. As is, the manuscript has 15 figures, which seems to detract from the strong mechanistic conclusion of the manuscript. Some figures are either negative data, or characterization of lines which are used. To consolidate figures would streamline the paper and make the mechanistic conclusion stand out. For example, Fig 13 and 14 could be combined, Figs. 11 and 12 could be compressed and combined, Figs. 5, 7,8 could be supplemental, Figs 9 and 10 could be combined and/or supplemental.
3. Labels in Fig. 1A should match description on page3.
4. Fig. 2A (and several others) would benefit from more detailed labeling as some areas referred to are not clear.
5. Typo on line 173-174 'indicates that' written twice.
6. Tense (past or present) of the results changes throughout manuscript.
7. There is some discrepancy in naming of transgenic lines. For example, the Wnt1-Cre animals are referred to as NCC-Fgfr2-/-, but the Scx-Cre;Fgfr2-/- follows more conventional naming.
8. Scx RNAscope in Fig. 4A is not that convincing, consider using the Scx-GFP to document the expression.
9. Labeling of Fig. 6 is confusing. Is the comparison a Sxc-GFP vs. NCC-Fgfr2-/-;Sxc-GFP? If so labeling the figure accordingly would clear that up.

First revision

Author response to reviewers' comments

We would like to thank the reviewers for their careful assessment of this manuscript. The comments provided were constructive and thoughtful. We have carefully addressed each recommendation as detailed below and believe this revised version is considerably stronger.

Reviewer 1:

1. *In several cases, the authors might consider additional arrows to point out specific structures.*

© 2024. Published by The Company of Biologists under the terms of the Creative Commons Attribution License (https://creativecommons.org/licenses/by/4.0/).

a. *The "cartilaginous fusions that follow a joint-gap pattern" in controls in Figure 1 I, should be indicated with an arrow or similar.*
b. *Figure 2 might benefit from arrows that point to ectocranial and endocranial domains, secondary osteogenic fronts, and bone fronts in general, as the bone front is not obvious without careful examination.*
c. *In Figure 6A-F, it might be helpful to mark with arrows the SCX and SCX/RUNX2 cells that are being referred to in the text. In this figure, higher magnification views of the bone fronts would also be helpful.*
d. *In Figure 8C and D the distinct Sox9 lineage cells that are being described in the text (endocranial AF/frontal bone, and endocranial domain of the PFS) might be highlighted with arrows for readers.*

Response: These suggested additions to the figures have been made.

2. *There is a typo on line 132; I believe it should read "posterior frontal".*
Response: This error has been corrected.

3. *Typo: on line 174 "indicates that" is duplicated.*
Response: This error has been corrected.

4. *On line 186, the authors indicate that "loss of Fgfr2 leads to a reduction of cells with the AF2 and OF2 identity. This seems potentially overstated, as this finding is based only on the expression of very few markers, which might be altered while cells remain present. The ideal data would be scRNA-seq on Fgfr2 mutants, but that would be quite a lot of work- can bulk RNA-seq data be used to further support this conclusion?*
Response: Thank you for this helpful suggestion. We have replaced the statement on line 186 with a more appropriate interpretation. On lines 298-305 and in Supplementary Figure 5, we have included an analysis that compares differentially expressed genes identified in the bulk RNA-seq with cluster enriched genes identified in the scRNA-seq dataset. These results suggest that genes associated with the AF clusters are upregulated, while those that are associated with the osteogenic clusters are downregulated.

5. *At line 745 (249), the authors suggest that "a small decrease in the proliferation rate of AF connective tissue could contribute to AF patency…" But it is not clear to me how a decrease in proliferation in the AF population fits with the observation of expanded AF- if proliferation contributed, wouldn't it be expected that these cells would have increased proliferation? Clarification would be helpful.*
Response: Thank you. We agree that our rationale was not clearly stated. We have now explained that either decreased proliferation in the OF or increased proliferation in the AF could be the cause of the phenotype. Since we only observed a slight decreased in AF proliferation, we do not believe that this difference in proliferation causes the persistent AF phenotype.

6. *While the rescue of Fgfr2 loss with Wif1 injection appears impressive, rescue experiments in Figure 14 should include some sort of quantitative and statistical analysis to reflect the extent and consistency of rescue across the n=7 replicates compared with mock injection controls.*
Response: This has now been included (New Figure 9G).

7. *In the discussion, the authors suggest that a similar etiology may exist for persistent AF patency in FGFR2 GOF, but it is not clear what is meant by this. Obviously, examination of AF phenotypes in GOF models is beyond the scope of this study, but further explanation of what is meant, and how these apparently opposite impacts on FGFR signaling could possibly result in the same AF phenotype is warranted in general, further discussion comparing the findings of this study with those that have investigated mechanisms of suture closure in Fgfr GOF synostosis models (e.g. is WNT signaling known to be impacted in those models?) would be of interest to better understand the scope and context of the mechanism that is uncovered here.*
Response: This has now been better addressed in the Discussion.

© 2024. Published by The Company of Biologists under the terms of the Creative Commons Attribution License (https://creativecommons.org/licenses/by/4.0/).

Reviewer 2:

Major suggestions:

1. *The various levels of expression of FgfR2 in different cell populations is interesting and begs the question of thresholds of Fgf activity (e.g., high in OF1/2 but low in AF1/2). Is there any evidence of 'high' or 'low' FGF activity priming cells for different fate decisions, as is the case for Wnt? If so, this may be a topic for discussion.*
   Response: This is a very interesting idea. Published studies have shown that FGF/ERK signaling dynamics control pluripotency in cultured mouse ESCs and cell specification in the mouse blastocyst. I am not aware of evidence for this in the context of the sutures or development of other complex organ systems.

2. *Which isoform of Fgfr2 is being expressed in these cell populations? Is it the same isoform in all populations? Given the variability and concordant functional implications, it might be worthwhile to explore isoform expression.*
   Response: Prior research in the suture and other developing organ systems has shown that the *Fgfr2-IIIc* splice variant is expressed by mesenchymal cells (including those in the frontal and coronal suture) and the Fgfr2-IIIb splice variant expressed by epithelial cells. A prior study has shown that selective knockout of the *Fgfr2-IIIc* splice variant in mice disrupts coronal suture development (Eswarakumar et al., 2002). The RNAScope probe we used in Figure 3D is specific for the *Fgfr2-IIIc* isoform. We have now indicated this in the Figure 2, its legend, and the text.

3. *Is Fgf18 the only ligand in the area? Some conclusions may be overstated regarding Fgfr2-Fgf18 if other ligands are expressed.*
   Response: The scRNA-seq dataset identified expression of *Fgf2*, *Fgf9*, and *Fgf18* in the EM and AF, which are all ligands for Fgfr2-IIIc. This is consistent with prior studies (Rice et al., 2000; Kim et al., 1998; Ohbayashi et al., 2002). We were unable to detect *Fgf9* using RNAScope and *Fgf2* was more generally distributed throughout the EM and AF. We focused on *Fgf18* because prior research has shown that loss of *Fgf18* in mice results in a persistent AF phenotype. Suture phenotypes have not been described in *Fgf2* and *Fgf9* knockout mice. Nevertheless, we agree that our analysis and interpretation should be less focused on *Fgf18*. We have now 1) included *Fgf2* and *Fgf9* in the violin plots in Figure 3B, 2) Included the CellChat analysis for the FGF signaling pathway network (replacing the FGF18-FGFR2 CellChat analysis), and 3) generalized our model.

4. *Is the repression of Wnt direct? If so, what is the mechanism?*
   Response: Our results suggests that decreased FGFR2 signaling in the OF leads to decreased expression of WIF1 in those cells. We believe that WIF1, which inhibits WNT signaling by binding to WNT proteins, is blocking WNT signaling in Fzd1-expressing cells in the AF. We do not yet know if FGFR2-mediated regulation of *Wif1* expression is direct.

5. *Has the lack of expression of Fgfr2 been confirmed through RNAscope in the NCC-Fgfr2-/- samples?*
   Response: The Fgfr2-flx allele used here was originally made in the lab of David Ornitz. This allele is known to produce a truncated transcript/protein that lacks the tyrosine kinase domain, and it is therefore a functional null (Yu et al., 2003, Development). In a prior study, we confirmed that the Orntiz allele generates a truncated transcript/protein when crossed to Wnt1-Cre (Roberts et al., 2019). In the AF, we find that the FGFR2 antibody and *Fgfr2-IIIc* RNAScope probe detect the truncated protein/transcript in the Wnt1-Cre;Fgfr2-flx/flx mice. We did not include this data in the manuscript, as it we do not believe it adds to the interpretation of the phenotype. The craniofacial phenotype we have identified using Wnt1-Cre and the Orntiz allele phenocopies that found when Wnt1-Cre is crossed to a null Fgfr2-flx allele generated by Philip Soriano.

© 2024. Published by The Company of Biologists under the terms of the Creative Commons Attribution License (https://creativecommons.org/licenses/by/4.0/).

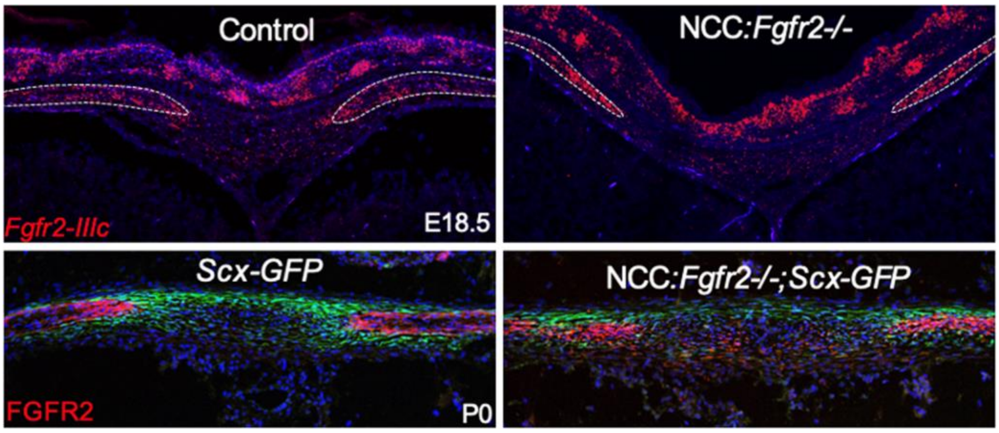

Minor:

1. *The current title seems broad and does not highlight the uncovered mechanism. Consider revising to incorporate the Wnt dependent mechanism.*
   Response: Thank you for this suggestion. We have changed the title to include WNT.

2. *As is, the manuscript has 15 figures, which seems to detract from the strong mechanistic conclusion of the manuscript. Some figures are either negative data, or characterization of lines which are used. To consolidate figures would streamline the paper and make the mechanistic conclusion stand out. For example, Fig 13 and 14 could be combined, Figs. 11 and 12 could be compressed and combined, Figs. 5, 7,8 could be supplemental, Figs 9 and 10 could be combined and/or supplemental.*
   Response: We have combined some figures (old figures 9/10, 13/15) and move Figures to supplemental (old Figures 5, 7, and 8).

3. *Labels in Fig. 1A should match description on page 3.*
   Response: This has been corrected.

4. *Fig. 2A (and several others) would benefit from more detailed labeling as some areas referred to are not clear.*
   Response: We have outlined the bone, and marked the endo- and ecto-cranial domains and cartilage in Figure 2A. We have also included annotations to better indicate the regions discussed in Figures 1, 6, and 8.

5. *Typo on line 173-174 'indicates that' written twice.*
   Response: This has been corrected.

6. *Tense (past or present) of the results changes throughout manuscript.*
   Response: This has been corrected.

7. *There is some discrepancy in naming of transgenic lines. For example, the Wnt1-Cre animals are referred to as NCC-Fgfr2-/-, but the Scx-Cre;Fgfr2-/- follows more conventional naming.*
   Response: We have change NCC-Fgfr2-/- to Wnt1-Cre;Fgfr2-/- throughout.

8. *Scx RNAscope in Fig. 4A is not that convincing, consider using the Scx-GFP to document the expression.*
   Response: This has now been included in Figure 4 (new panel C and D).

9. *Labeling of Fig. 6 is confusing. Is the comparison a Scx-GFP vs. NCC-Fgfr2-/-;Scx-GFP? If so, labeling the figure accordingly would clear that up.*
   Response: This has been corrected.

© 2024. Published by The Company of Biologists under the terms of the Creative Commons Attribution License (https://creativecommons.org/licenses/by/4.0/).

Second decision letter

MS ID#: dev.204264R1

MS TITLE: FGFR2 Directs Inhibition of WNT Signaling to Regulate Anterior Fontanelle Closure during Skull Development

AUTHORS: Lauren Bobzin; Audrey Nickle; Sebastian Ko; Michaela Ince; Aaron Huang; Arshia Bhojwani; Ryan Roberts; Amy E. Merrill

I am happy to tell you that your manuscript has been accepted for publication in Development, pending our standard publication integrity checks.

Comments from the Reviewers:

Reviewer 1: SUMMARY OF THE ADVANCE MADE IN THIS PAPER AND ITS POTENTIAL SIGNIFICANCE TO THE FIELD

The authors have addressed concerns raised in the first revision. This is a very nice study that advances our understanding of the role of FGFR signaling in calvarial development.

SUGGESTIONS TO AUTHORS

Reviewer 2: SUMMARY OF THE ADVANCE MADE IN THIS PAPER AND ITS POTENTIAL SIGNIFICANCE TO THE FIELD

SUGGESTIONS TO AUTHORS
Authors have addressed all my concerns.

© 2024. Published by The Company of Biologists under the terms of the Creative Commons Attribution License (https://creativecommons.org/licenses/by/4.0/).
